# CRISPR-Cas9 bends and twists DNA to read its sequence

**DOI:** 10.1038/s41594-022-00756-0

**Published:** 2022-04-14

**Authors:** Joshua C. Cofsky, Katarzyna M. Soczek, Gavin J. Knott, Eva Nogales, Jennifer A. Doudna

**Affiliations:** 1 Department of Molecular and Cell Biology, University of California, Berkeley, Berkeley, California, USA.; 2 California Institute for Quantitative Biosciences (QB3), University of California, Berkeley, Berkeley, California, USA.; 3 Innovative Genomics Institute, University of California, Berkeley, Berkeley, California, USA.; 4 Monash Biomedicine Discovery Institute, Department of Biochemistry & Molecular Biology, Monash University, VIC 3800, Australia.; 5 Howard Hughes Medical Institute, University of California, Berkeley, Berkeley, California, USA.; 6 MBIB Division, Lawrence Berkeley National Laboratory, Berkeley, Berkeley, California, USA.; 7 Department of Chemistry, University of California, Berkeley, Berkeley, California, USA.; 8 Gladstone Institutes, University of California, San Francisco, San Francisco, California, USA.

## Abstract

In bacterial defense and genome editing applications, the CRISPR-associated protein Cas9 searches millions of DNA base pairs to locate a 20-nucleotide, guide-RNA-complementary target sequence that abuts a protospacer-adjacent motif (PAM). Target capture requires Cas9 to unwind DNA at candidate sequences using an unknown ATP-independent mechanism. Here we show that Cas9 sharply bends and undertwists DNA upon PAM binding, thereby flipping DNA nucleotides out of the duplex and toward the guide RNA for sequence interrogation. Cryo-electron-microscopy (EM) structures of Cas9:RNA:DNA complexes trapped at different states of the interrogation pathway, together with solution conformational probing, reveal that global protein rearrangement accompanies formation of an unstacked DNA hinge. Bend-induced base flipping explains how Cas9 “reads” snippets of DNA to locate target sites within a vast excess of non-target DNA, a process crucial to both bacterial antiviral immunity and genome editing. This mechanism establishes a physical solution to the problem of complementarity-guided DNA search and shows how interrogation speed and local DNA geometry may influence genome editing efficiency.

## Introduction

CRISPR-Cas9 (clustered regularly interspaced short palindromic repeats, CRISPR-associated) nucleases provide bacteria with RNA-guided adaptive immunity against viral infections^1^ and serve as powerful tools for genome editing in human, plant and other eukaryotic cells^2^. The basis for Cas9’s utility is its DNA recognition mechanism, which involves base-pairing of one DNA strand with 20 nucleotides of the guide RNA to form an R-loop. The guide RNA’s recognition sequence, or “spacer,” can be chosen to match a desired DNA target, enabling programmable site-specific DNA selection and cutting^3^. The search process that Cas9 uses to comb through the genome and locate rare target sites requires local unwinding to expose DNA nucleotides for RNA hybridization, but it does not rely on an external energy source such as ATP hydrolysis^4,5^. This DNA interrogation process defines the accuracy and speed with which Cas9 induces genome edits, yet the mechanism remains unknown.

**Fig. 1 |:**
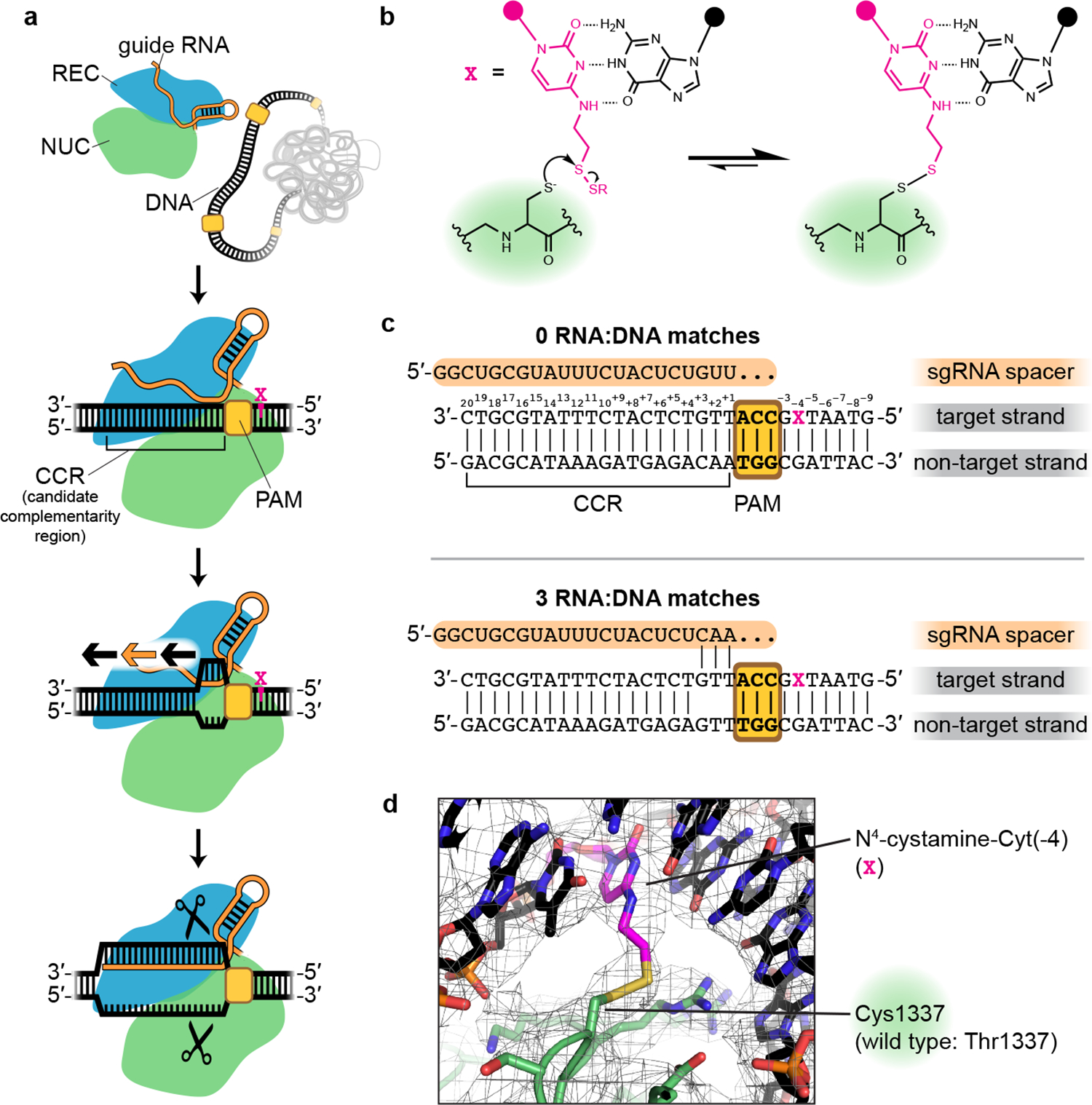
Trapping the Cas9 interrogation complex. **a**, Known steps leading to Cas9-catalyzed DNA cleavage. Orange/black arrows indicate direction of guide RNA strand invasion into the DNA helix. Magenta X indicates location of cystamine modification. **b**, Chemistry of protein:DNA cross-link. **c**, RNA and DNA sequences used in structural studies. **d**, Sharpened cryo-EM map (threshold 7σ) and model of the cross-linked Cas9:sgRNA:DNA complex (0 RNA:DNA matches, bent DNA), centered on density contributed by the non-native thioalkane cross-link.

Molecular structures of Cas9^6^ in pre- and post-DNA bound states revealed that the protein’s REC (“recognition”) and NUC (“nuclease”) lobes can rotate dramatically around each other, assuming an “open” conformation in the apo Cas9 structure^7^ and a “closed” conformation in the Cas9:guide-RNA^8,9^ and Cas9:guide-RNA:DNA R-loop^9^ structures. Furthermore, single-molecule experiments established the importance of PAMs (5′-NGG-3′) for pausing at candidate targets^5,10^, and R-loop formation was found to occur through directional strand invasion beginning at the PAM^5^ (Fig. 1a). However, these findings did not elucidate the actions that Cas9 performs to interrogate each candidate target sequence. These actions, repeated over and over, comprise the slowest phase of Cas9’s bacterial immune function and its induction of site-specific genome editing^11^. Understanding the mechanism of DNA interrogation is critical to determining how Cas9 searches genomes to find *bona fide* targets and exclude the vast excess of non-target sequences.

## Results and Discussion

### Covalent cross-linking of Cas9 to DNA stabilizes the interrogation complex

Evidence that Cas9’s target engagement begins with PAM binding^5^ implies that during genome search, there exists a transient “interrogation state” in which Cas9:guide RNA has engaged with a PAM but not yet formed RNA:DNA base pairs (Fig. 1a). Cas9:guide-RNA complexes must repeatedly visit the interrogation state at each surveyed PAM, irrespective of the sequence of the adjacent 20-base-pair (bp) candidate complementarity region (CCR). While this state is the key to Cas9’s DNA search mechanism, the interrogation complex has so far evaded structure determination due to its transience, with an estimated lifetime of <30 milliseconds in bacteria^11^.

**Extended Data Fig. 1:**
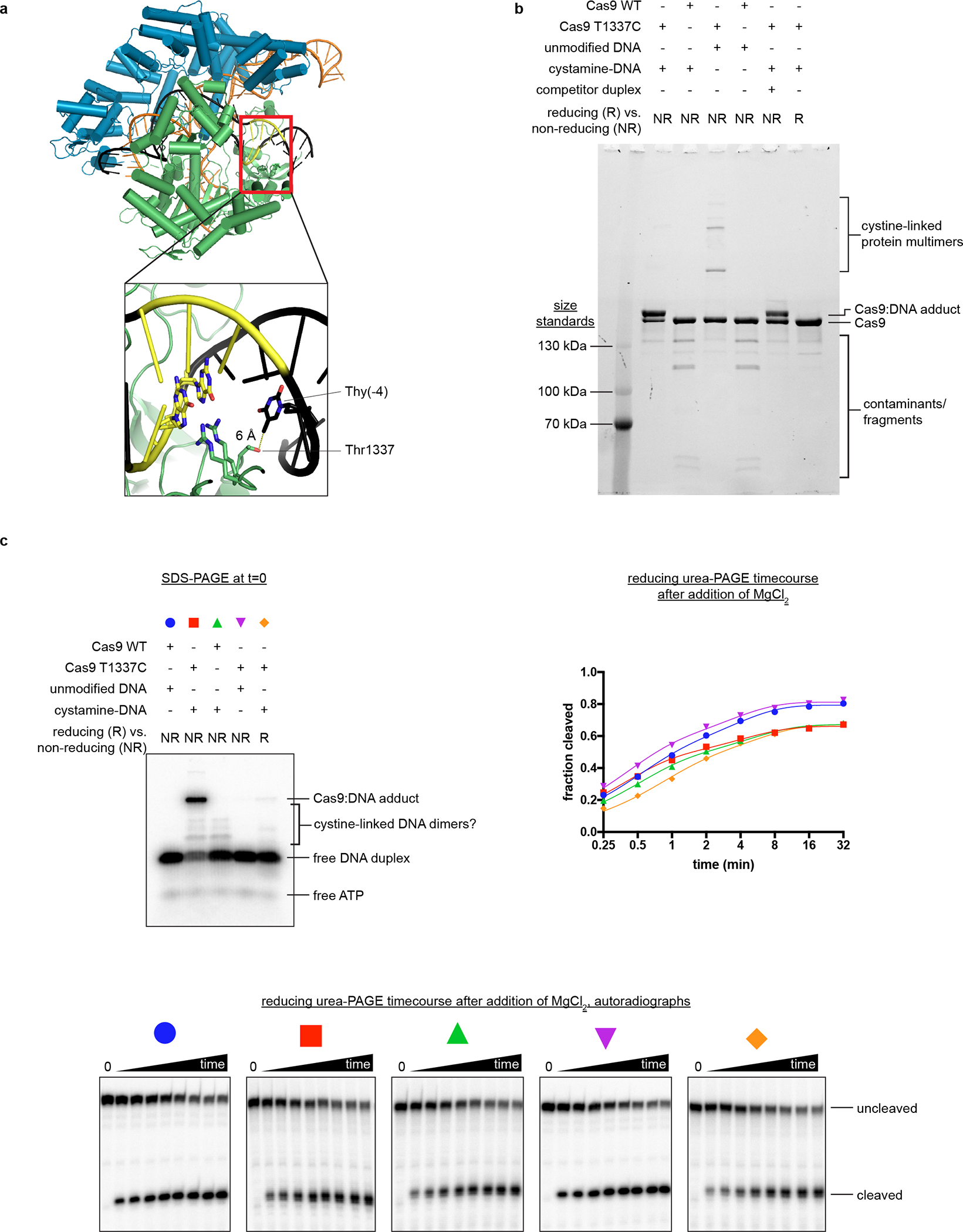
Characterization of the Cas9:DNA cross-link. **a**, Crystal structure of Cas9:sgRNA:DNA with 20-bp RNA:DNA hybrid formed (PDB 4UN3). In the inset, Arg1333 and Arg1335 recognize the two guanines of the PAM. Green, NUC lobe; blue, REC lobe; orange, guide RNA; black, DNA; yellow, PAM. **b**, Non-reducing SDS-PAGE (Stain-Free) analysis of cross-linking reactions and controls. Complexes were prepared identically to structural constructs but in smaller volumes and without size exclusion purification. Competitor duplex, where indicated, was added before the cross-linkable duplex at an equivalent concentration. The depicted experiment was performed once, and a prior optimization experiment yielded similar results. **c**, Top left, non-reducing SDS-PAGE autoradiograph to determine the fraction of DNA cross-linked to Cas9 at t=0. The target strand is radiolabeled. Bottom, reducing urea-PAGE autoradiograph revealing target-strand cleavage kinetics; quantification depicted in top right. The depicted model is $y=C\left(1-e^{-k_{1}t}\right)+(B_{max}-C)(1-e^{-k_{2}t})$. The depicted experiments were performed once, and a prior optimization experiment yielded similar results.

To trap the Cas9 interrogation complex, we replaced residue Thr1337 with cysteine in *S. pyogenes* Cas9, the most widely used genome editing enzyme, and combined this protein with a single-guide RNA (sgRNA)^3^ and a 30-bp DNA molecule functionalized with an N^4^-cystamine cytosine modification^12^ (Fig. 1b). The DNA included a PAM but lacked any complementarity to the sgRNA spacer (Fig. 1c). Reaction of the cysteine thiol with the cystamine creates a protein:DNA disulfide cross-link on the side of the PAM distal to the site of R-loop initiation (Fig. 1b,d). The position of the cross-link was chosen based on previous high-resolution structures of the Cas9:PAM interface^9,13^ (Extended Data Fig. 1a). Incubation of Cas9 T1337C with sgRNA and the modified DNA duplex resulted in a decrease in electrophoretic mobility for ~70% of the total protein mass under denaturing but non-reducing conditions (Extended Data Fig. 1b), consistent with protein:DNA cross-link formation. The cross-link did not inhibit Cas9’s ability to cleave sgRNA-complementary DNA (Extended Data Fig. 1c), suggesting that the enzyme is not grossly perturbed by the introduced disulfide. More importantly, mechanistic hypotheses revealed by cross-linked complexes can be tested in non-cross-linked complexes.

**Extended Data Fig. 2:**
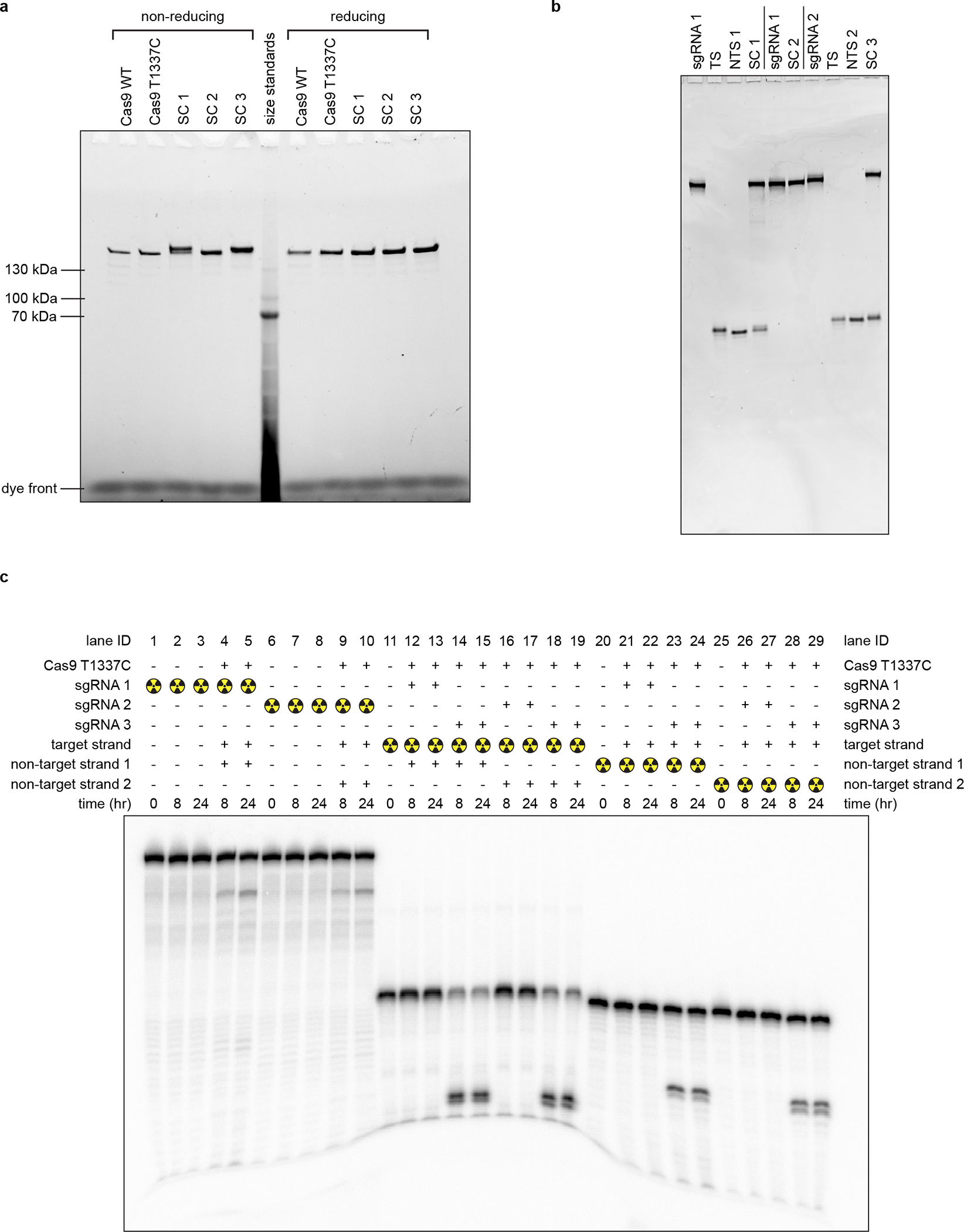
Cryo-EM sample quality. **a**, SDS-PAGE (Stain-Free) analysis of purified proteins and cryo-EM samples. SC, structural construct; SC 1, Cas9:sgRNA:DNA with 0 RNA:DNA matches; SC 2, Cas9:sgRNA; SC 3, Cas9:sgRNA:DNA with 3 RNA:DNA matches. The depicted experiment was performed once (on the samples used for the final cryo-EM grids). **b**, Reducing urea-PAGE (SYBR-Gold-stained) analysis of purified nucleic acid components and cryo-EM samples. TS, target strand; NTS, non-target strand. The depicted experiment was performed once (on the samples used for the final cryo-EM grids). **c**, Reducing urea-PAGE autoradiograph of radioactive mimics of structural constructs. sgRNA 1 and non-target strand 1 are those used to create SC 1. sgRNA 2 and non-target strand 2 are those used to create SC 3. sgRNA 3 bears a spacer with 20 nt of complementarity to the DNA target strand. The depicted experiment was performed once, and a prior optimization experiment yielded similar results.

**Extended Data Fig. 3:**
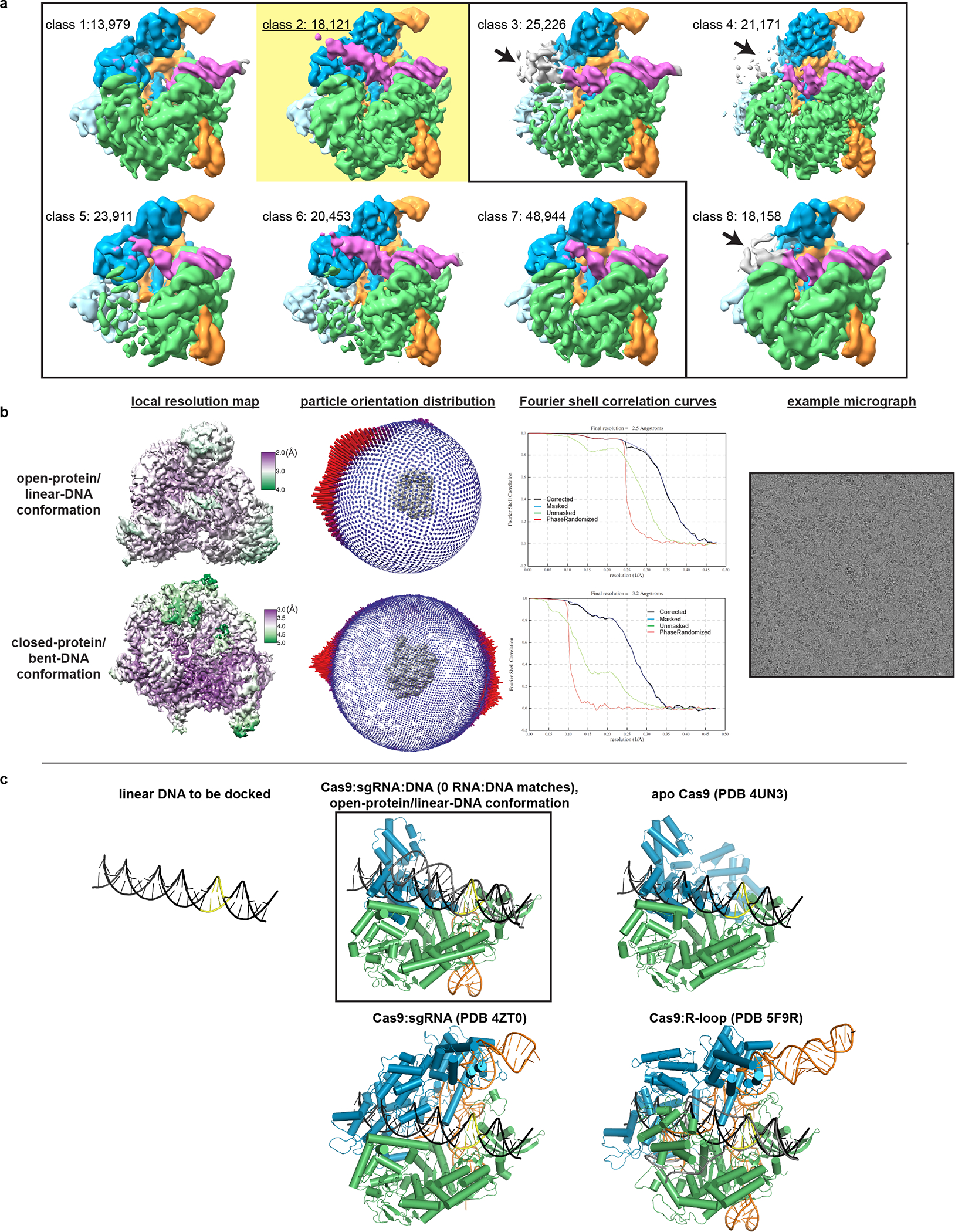
Cryo-EM analysis of Cas9:sgRNA:DNA with 0 RNA:DNA matches. **a**, Classes from RELION 3D classification of closed-protein particles (threshold 6σ). The number of particles in each class is indicated next to the class number. In classes 1/2/5/6/7 the DNA is bent next to the PAM (visible for class 1 at lower contour). In classes 3/4/8 the DNA continues along a more linear trajectory for half a turn past the PAM, into the region normally occupied by REC2; in these classes, density in the region of the putative collision (black arrow) is uninterpretable as either protein or DNA, likely due to particle damage, and is thus colored gray. The class used for the final closed-protein/bent-DNA map is class 2, highlighted in yellow. Green, NUC lobe; blue, REC lobe domains 1/2; light blue, REC lobe domain 3; orange, guide RNA; magenta, DNA. **b**, Details of final cryo-EM maps. **c**, Linear DNA docked into open-protein/linear-DNA cryo-EM structure and previous Cas9 crystal structures. All structures were aligned to the C-terminal domain of PDB 5F9R; then, the linear DNA was aligned to the PAM-containing duplex of PDB 5F9R. Green, NUC lobe; blue, REC lobe; orange, guide RNA; black, docked DNA; yellow, PAM. The DNA truly belonging to each structure (if present) is depicted in gray.

**Fig. 2 |:**
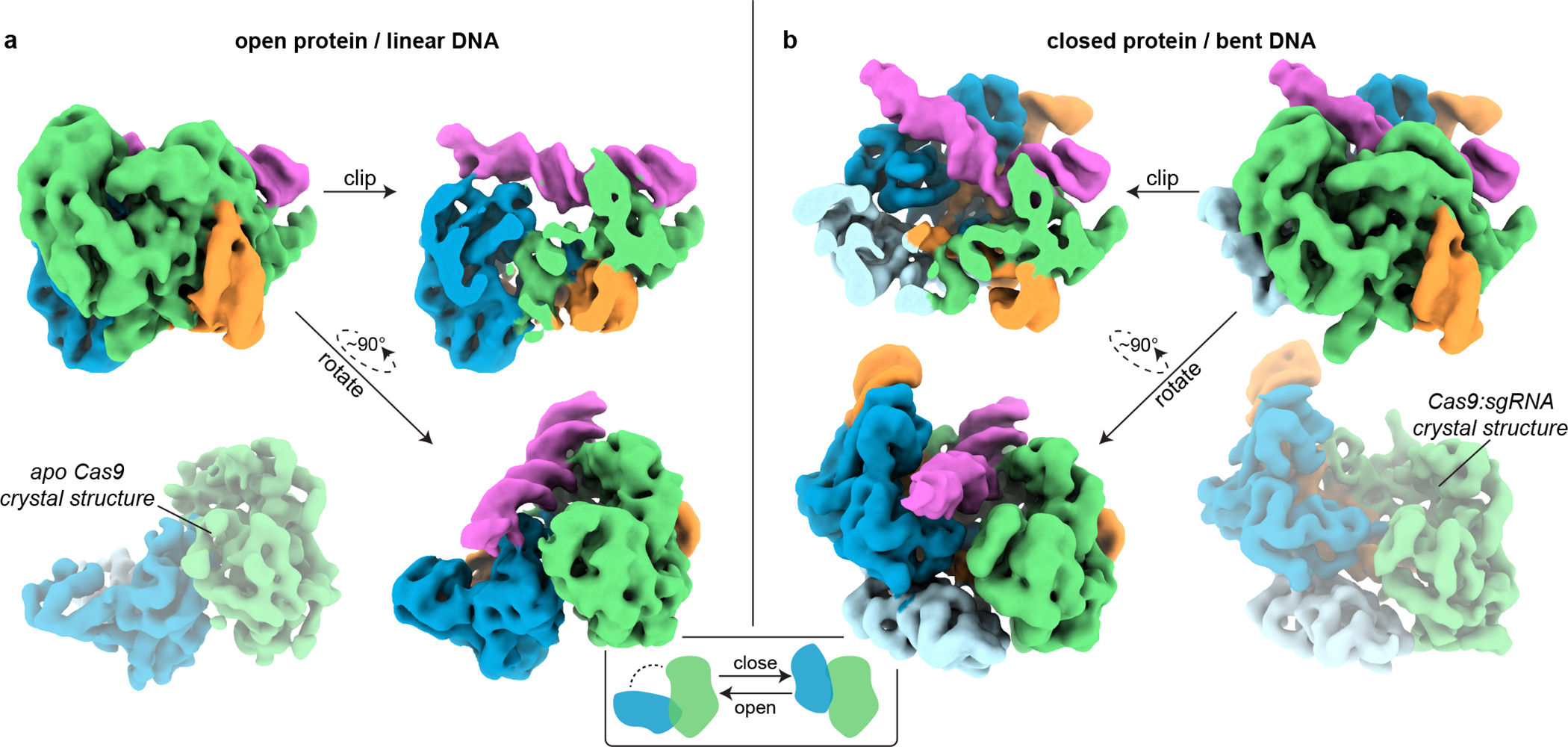
Cryo-EM structures of the Cas9 interrogation complex, compared to previously determined crystal structures. **a**, Unsharpened cryo-EM map (threshold 4σ) of Cas9 interrogation complex in open-protein/linear-DNA conformation, alongside apo Cas9 crystal structure (PDB 4CMP, 2Fo-Fc, threshold 1.5σ). **b**, Closed-protein/bent-DNA conformation (threshold 5σ), alongside Cas9:sgRNA crystal structure (PDB 4ZT0, 2Fo-Fc, threshold 1.5σ). Green, NUC lobe; blue, REC lobe domains 1/2; light blue, REC lobe domain 3; orange, guide RNA; magenta, DNA. Maps produced in the present work are displayed with full opacity. REC lobe domain 3 does not appear in the cryo-EM structure in **a** (see Supplementary Information). Additional classes observed in the closed-protein state are shown in Extended Data Fig. 3a.

We subjected the cross-linked interrogation complex to cryo-EM imaging and analysis (Extended Data Figs. 2, 3a–b). *Ab initio* volume reconstruction, refinement and modeling revealed two structural states of the complex. In one, the DNA lies as a linear duplex across the surface of the open form of the Cas9 ribonucleoprotein (Fig. 2a). Remarkably, in the other state, Cas9’s two lobes pinch the DNA into a V shape whose helical arms meet at the site of R-loop initiation, employing a bending mode that underwinds the DNA duplex (Fig. 2b).

### The linear-DNA conformation reveals a DNA scanning state of Cas9

In the “linear-DNA” conformation (Fig. 2a), the interface of the DNA with the PAM-interacting domain is similar to that seen in the crystal structure of Cas9:R-loop^9^ (Extended Data Fig. 3c). However, the REC lobe of the protein is in a position radically different from that observed in all prior structures of nucleic-acid-bound Cas9^8,9,13,14^, having rotated away from the NUC lobe into an open-protein conformation that resembles the apo Cas9 crystal structure^7^ (Fig. 2a).

**Extended Data Fig. 4:**
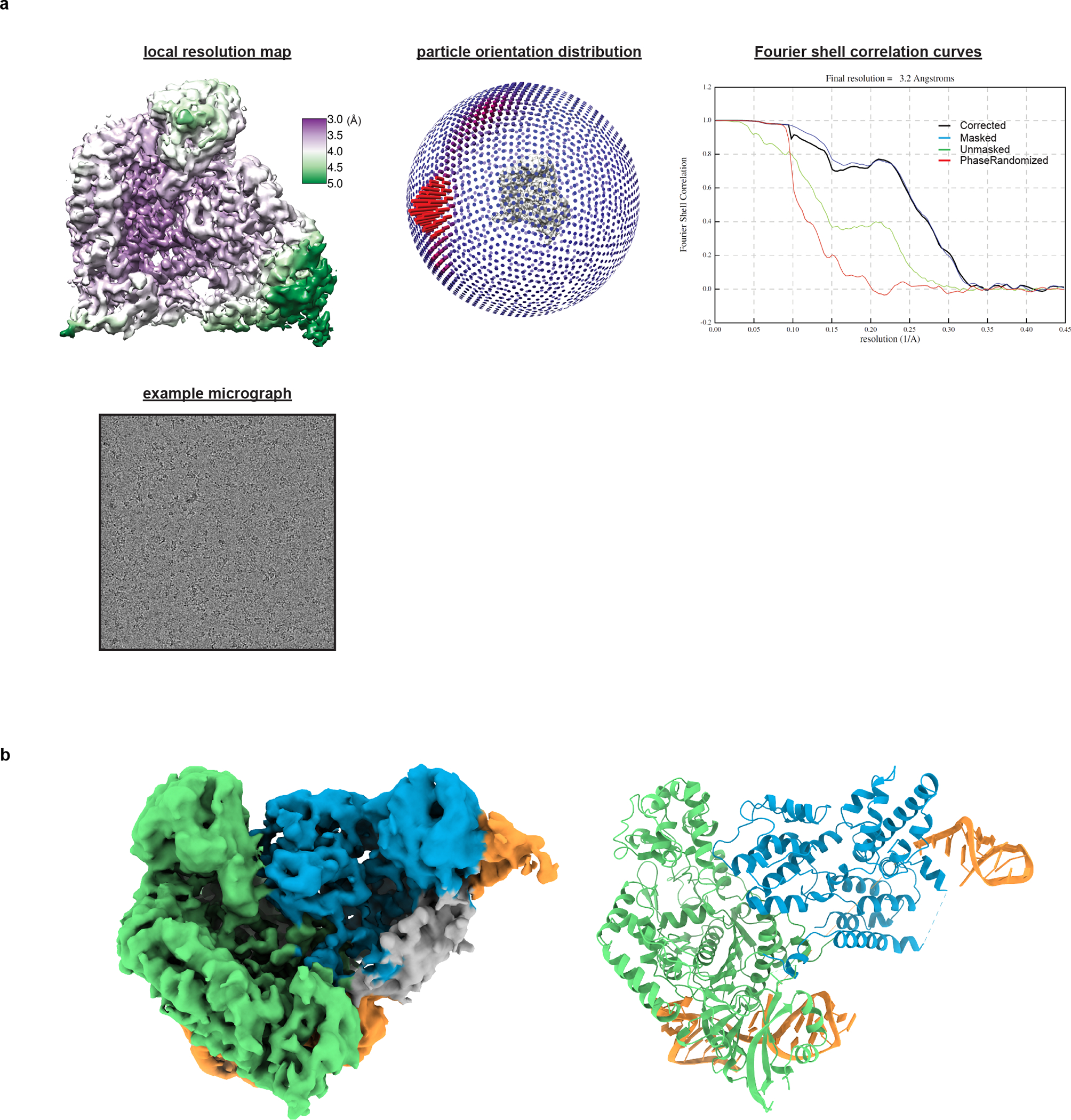
Cryo-EM analysis of Cas9:sgRNA. **a**, Details of cryo-EM analysis. **b**, Unsharpened cryo-EM map (threshold 5σ) and model of Cas9:sgRNA in open-protein conformation. Green, NUC lobe; blue, REC lobe; orange, guide RNA; gray, unattributed density (REC1 or guide RNA, see Supplementary Information).

Notably, a linear piece of DNA docked into the PAM-binding cleft would result in a severe structural clash^4^ in either the Cas9:R-loop^9^ or the Cas9:sgRNA^8^ crystal structure but only a minor one in the apo Cas9 crystal structure^7^, which can be relieved by slightly tilting and bending the DNA (Extended Data Fig. 3c). We propose that the open-protein conformation, originally thought to be unique to nucleic-acid-free Cas9, can also be adopted by the sgRNA-bound protein to enable its interaction with linear DNA. Indeed, cryo-EM analysis of the Cas9:sgRNA complex revealed only particles in the open-protein state (Extended Data Fig. 4), indicating that the original crystal structure of Cas9:sgRNA^8^, which was in a closed-protein state, represented only one possible conformation of the complex that happened to be captured in that crystal form. Single-molecule Förster resonance energy transfer experiments also support the ability of Cas9:sgRNA to access both closed and open conformations^15^. The linear-DNA/open-protein conformation captured in our cryo-EM structure, then, may represent the conformation of Cas9 during any process for which it must accommodate a piece of linear DNA, such as during sliding^10^ or initially engaging with a PAM.

### The bent-DNA conformation reveals PAM-adjacent DNA unwinding by Cas9

**Fig. 3 |:**
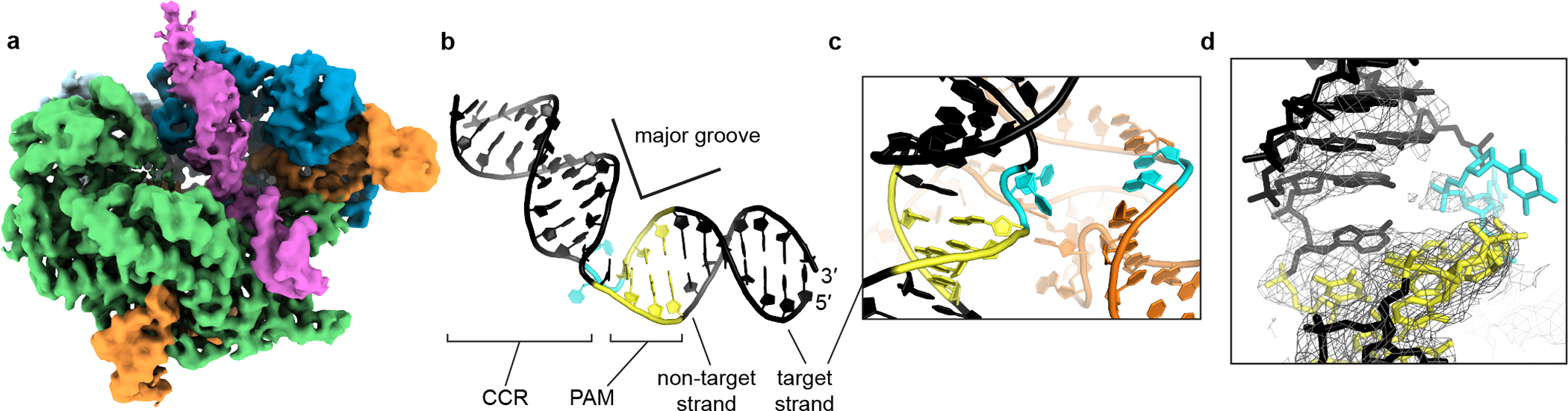
DNA conformation at the site of bending. **a**, Unsharpened cryo-EM map (threshold 5σ) of Cas9 interrogation complex in closed-protein/bent-DNA conformation. Green, NUC lobe; blue, REC lobe; orange, guide RNA; magenta, DNA. **b**, Bent DNA model. CCR, candidate complementarity region. Yellow, PAM; cyan, target-strand Thy(+1) and Thy(+2). **c**, DNA (black) and RNA (orange) models, demonstrating proximity of the first DNA:RNA base pairs (cyan) that would form if they were complementary. **d**, DNA model within sharpened cryo-EM density (threshold 7.5σ). The break in density in the target strand indicates dramatic conformational heterogeneity. The modeled conformation of Thy(+1) and Thy(+2) (cyan), which represents just one possible conformation within a diverse ensemble, was chosen based on permanganate reactivity data and geometric constraints imposed by neighboring nucleotides.

In the “bent-DNA” Cas9 interrogation complex, the protein grips the PAM as in the linear-DNA complex. The CCR (candidate complementarity region), on the other hand, is tilted at a 50° angle to the PAM-containing helix and leans against the REC lobe, which has risen into the same “closed” position as in the Cas9:sgRNA crystal structure (Fig. 2b, 3a). Compared to the high-resolution cryo-EM density contributed by the PAM-containing duplex, CCR density is poorly resolved (Fig. 3a, Extended Data Fig. 3b), reflecting conformational heterogeneity; however, even at low resolution, its distinct helical shape (Fig. 2b, 3a) enabled construction of an atomic model that adheres to B-form DNA constraints between the PAM-distal tip and position +3 of the CCR (Fig. 3b). Connection of this B-form helix to the PAM-containing helix requires backbone distortion and helical underwinding at precisely the position from which an R-loop would be initiated if the sgRNA were complementary to the CCR^5^ (Fig. 3b,c). Underwinding is accomplished through major groove compression (Fig. 3b), as observed for other protein-induced bends^16–23^.

At the distorted bending vertex, a missing wedge of cryo-EM density appears across from non-target-strand nucleotides Ade(+1) and Ade(+2), suggesting that target-strand nucleotides Thy(+1) and Thy(+2) have become unpaired from their partners (Fig. 3d). The overall weakness of density for Thy(+1) and Thy(+2) suggests dramatic mobility, and the modeled conformation of those nucleotides represents a physically plausible member of a diverse conformational ensemble (which also agrees with solution experiments to be discussed shortly). Therefore, in the bent-DNA conformation, two helical arms join at an underwound hinge whose target-strand nucleotides are heterogeneously positioned. DNA disorder is consistent with the function of the Cas9 interrogation complex, which is to flip target-strand nucleotides from the DNA duplex toward the sgRNA to test base pairing potential.

### Cas9:sgRNA bends DNA in non-cross-linked complexes

**Extended Data Fig. 5:**
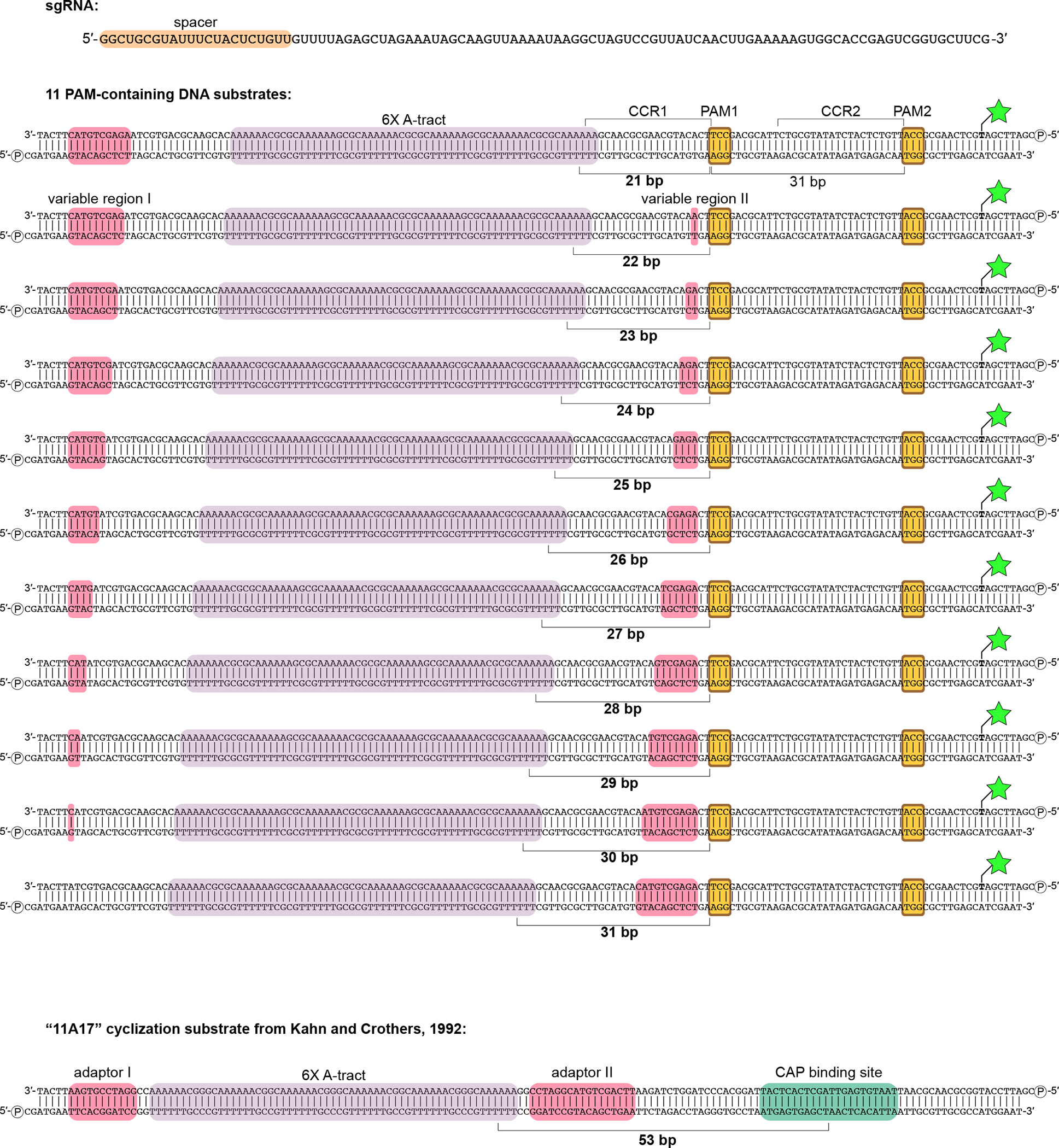
Nucleic acid sequences used in DNA cyclization experiments. Green star/bold T, fluorescein-conjugated dT; circled P, 5′ phosphate; CCR, candidate complementarity region.

**Extended Data Fig. 6:**
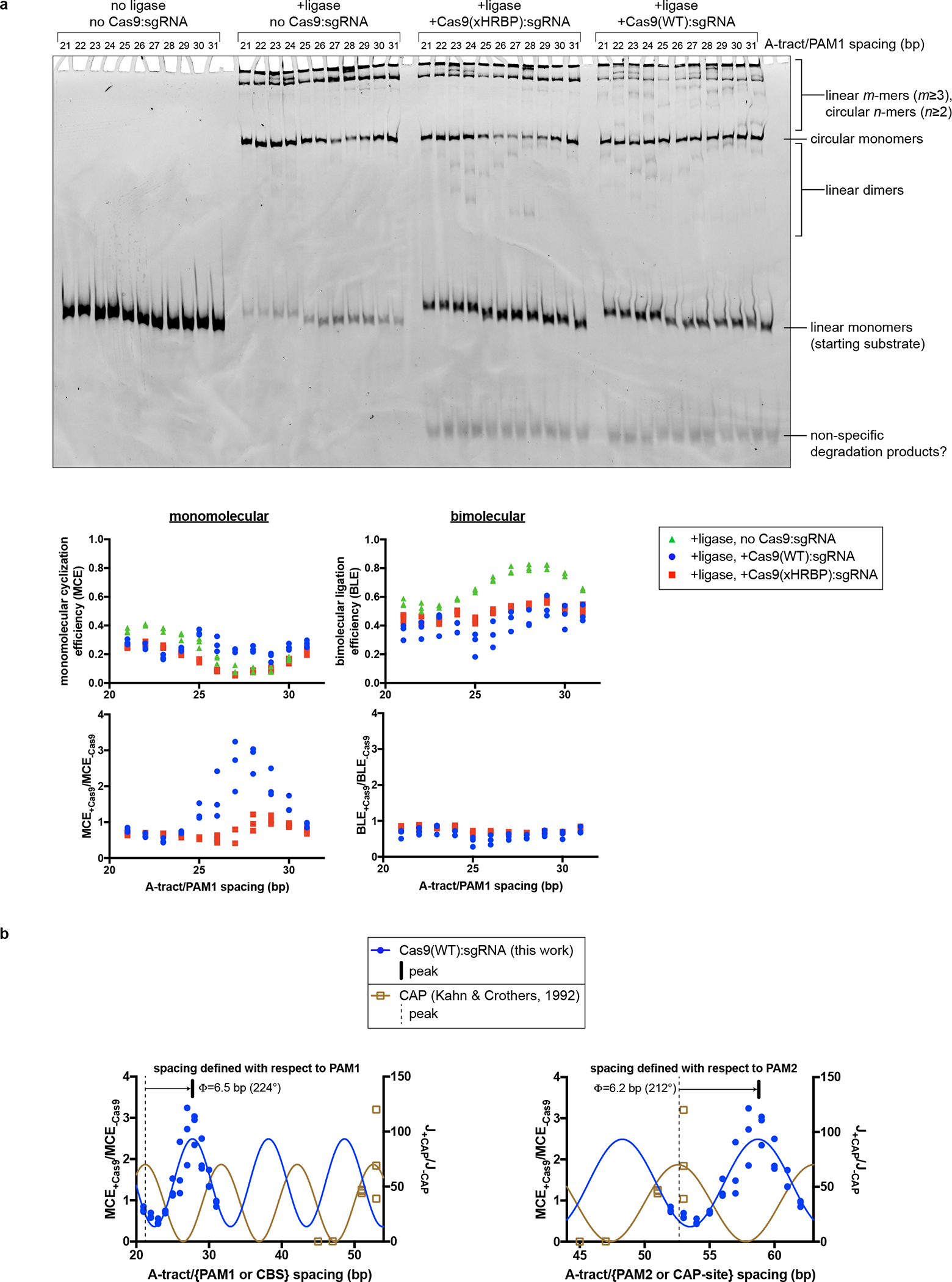
Details of DNA cyclization experiments. **a**, Fluorescence image and analysis of native PAGE gel resolving ligation products. Gel represents one replicate. Three replicates are plotted on the graphs. The polymeric/cyclized band assignments were made by reference to the relative electrophoretic mobilities observed in Kahn & Crothers, 1992. **b**, Comparison of Cas9:DNA cyclization data to CAP:DNA cyclization data. The depicted model is $y=A\cdot \sin\left(\frac{2\pi }{10.45\;bp}\left(x+\phi_{0}\right)\right)+b$, with the following constraints: A>0,b>A. The average of 224° and 212° is reported in Fig. 4c. J, J-factor (defined in Kahn & Crothers, 1992); Φ, phase difference; CBS, CAP-binding site.

**Fig. 4 |:**
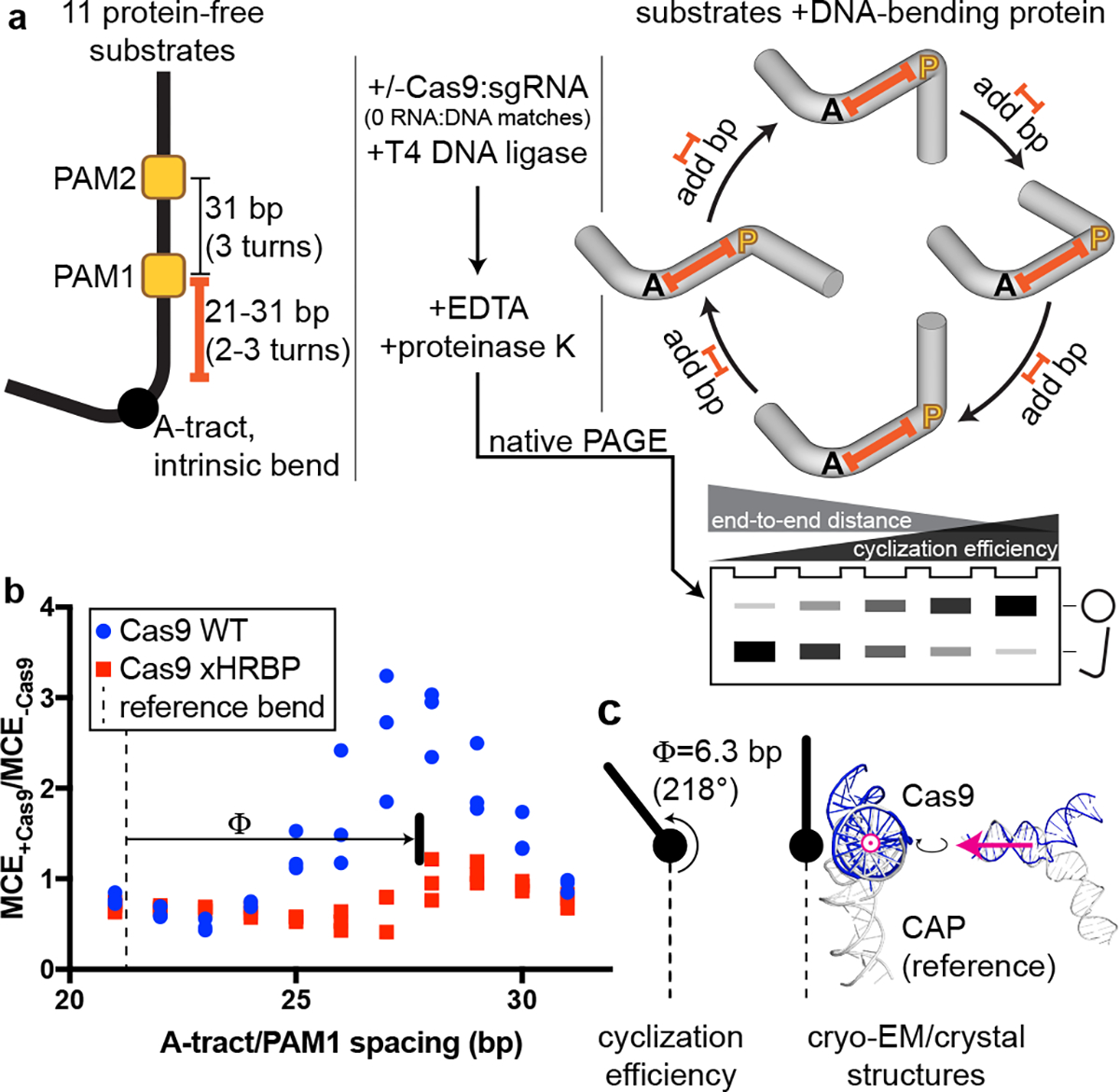
DNA cyclization efficiency experiments. **a**, Substrate structure and experimental pipeline. Black A, A-tract; yellow P, PAM; orange spacer, A-tract/PAM1 distance (21–31 bp). For simplicity, bending is only depicted at a single PAM in the cylindrical volume illustrations. Substrates that are more S-shaped (left side of the cycle diagram/gel) cyclize more slowly than substrates that are C-shaped (right) due to changes in the end-to-end distance of the molecules. When base pairs are added to the spacer, the cyclization efficiency is expected to rise as the Cas9-induced bend becomes aligned with the A-tract bend, then fall as the bends become misaligned again, in a roughly sinusoidal pattern. **b**, Cas9-dependent cyclization enhancement of eleven substrate variants. Three replicates are depicted. MCE, monomolecular cyclization efficiency; xHRBP, mutated helix-rolling basic patch (K233A/K234A/K253A/K263A); Φ, phase difference from reference bend to the Cas9 WT peak. A protein that does not bend the DNA at all would yield the line y=1. A protein that bends DNA in a different direction would yield an x-shifted sinusoid that peaked at a different value of spacer length. **c**, Comparison of bending phase difference in the cyclization experiments vs. cryo-EM/crystal structures of DNA bends introduced by Cas9 or CAP (PDB 1CGP). See Supplementary Information for discussion of the CAP-based reference bend analysis. The magenta vector superposed on the aligned helices would point toward the A-tract in the cyclization substrates; in the larger structural diagram, it points out of the page.

To determine whether unmodified Cas9 can bend DNA, we produced interrogation complexes that lacked the cross-link and tested them in a DNA cyclization assay^24^ (Fig. 4a). We created a series of 160-bp double-stranded DNA substrates that all bore a “J”-shape due to the inclusion of a special A-tract sequence that forms a protein-independent 108° bend^25^. Each substrate also included two PAMs spaced by a near-integral number of B-form DNA turns (31 bp). In eleven versions of this substrate, we varied the number of base pairs between the A-tract and the proximal PAM from 21 to 31 bp, effectively rotating the Cas9 binding sites around an entire turn of a B-form DNA helix (Extended Data Fig. 5). If Cas9 bends the DNA, each additional base pair added to the variable (21–31 bp) region will turn the Cas9-induced bend by ~34° with respect to the fixed A-tract bend. The relative direction of the two bends can be discerned from each substrate’s ligase-catalyzed cyclization efficiency, which should increase when the two bends point in the same direction (DNA assumes a “C” shape) and decrease when the two bends point in opposite directions (DNA assumes an “S” shape), as a function of the proximity of the DNA ends to be sealed (Fig. 4a). We measured the cyclization efficiency of each substrate in the absence and presence of Cas9 and an sgRNA lacking homology to either of the two CCR sequences. Consistent with expectations for a bend, the Cas9-dependent enhancement (or reduction) of cyclization efficiency tracked a sinusoidal shape when plotted against the A-tract/PAM spacing, reflecting phase-dependent variation in the end-to-end distance of different substrates (Fig. 4b, Extended Data Fig. 6a, b). Additionally, by interpreting the absolute phase of the cyclization enhancement curve (that is, the spacing value at which the peak occurs, where the two bends point in the same direction) in the context of the known direction of the A-tract bend^24,26^, we conclude that the bending direction observed in this experiment is the same as that observed in the bent-DNA cryo-EM structure (Fig. 4c, Extended Data Fig. 6b, Supplementary Information).

**Extended Data Fig. 7:**
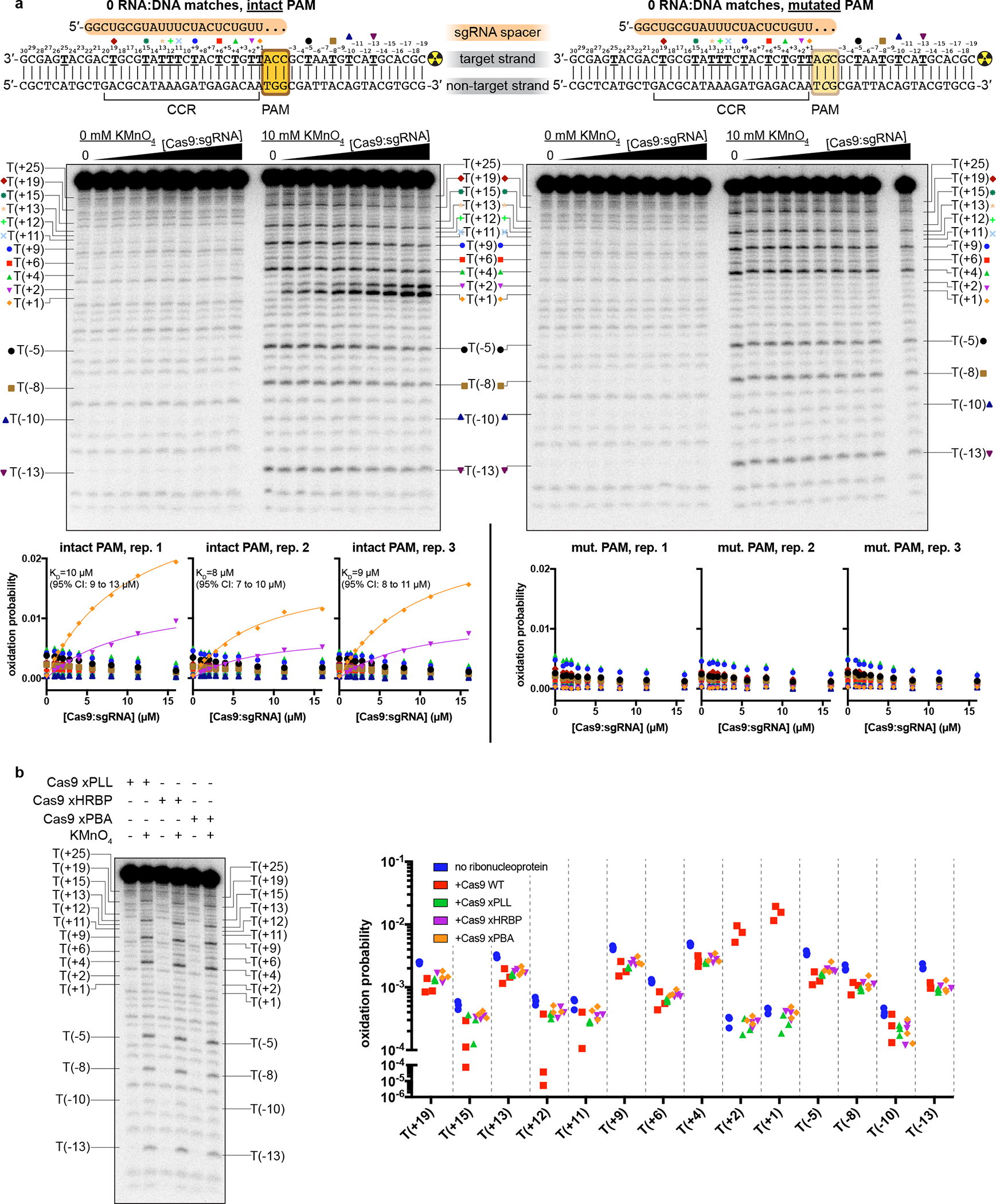
Details of permanganate reactivity measurements. **a**, Autoradiographs and analysis of all thymines except T(+25), which was insufficiently resolved from neighboring bands. The depicted autoradiographs are replicate 1. Due to systematic variation across replicates, individual replicates are presented on separate graphs and fitted separately. The depicted model is $p_{ox}=\frac{B_{max}[Cas9:sgRNA]}{K_{D}+[Cas9:sgRNA]}$, with KD shared across T(+1) and T(+2). CCR, candidate complementarity region; CI, confidence interval. **b**, Autoradiograph (same gel as “intact PAM” autoradiograph in **a**) and analysis of experiments containing variants of Cas9:sgRNA (16 μM), with the “intact PAM” DNA substrate. Graph depicts three replicates.

**Fig. 5 |:**
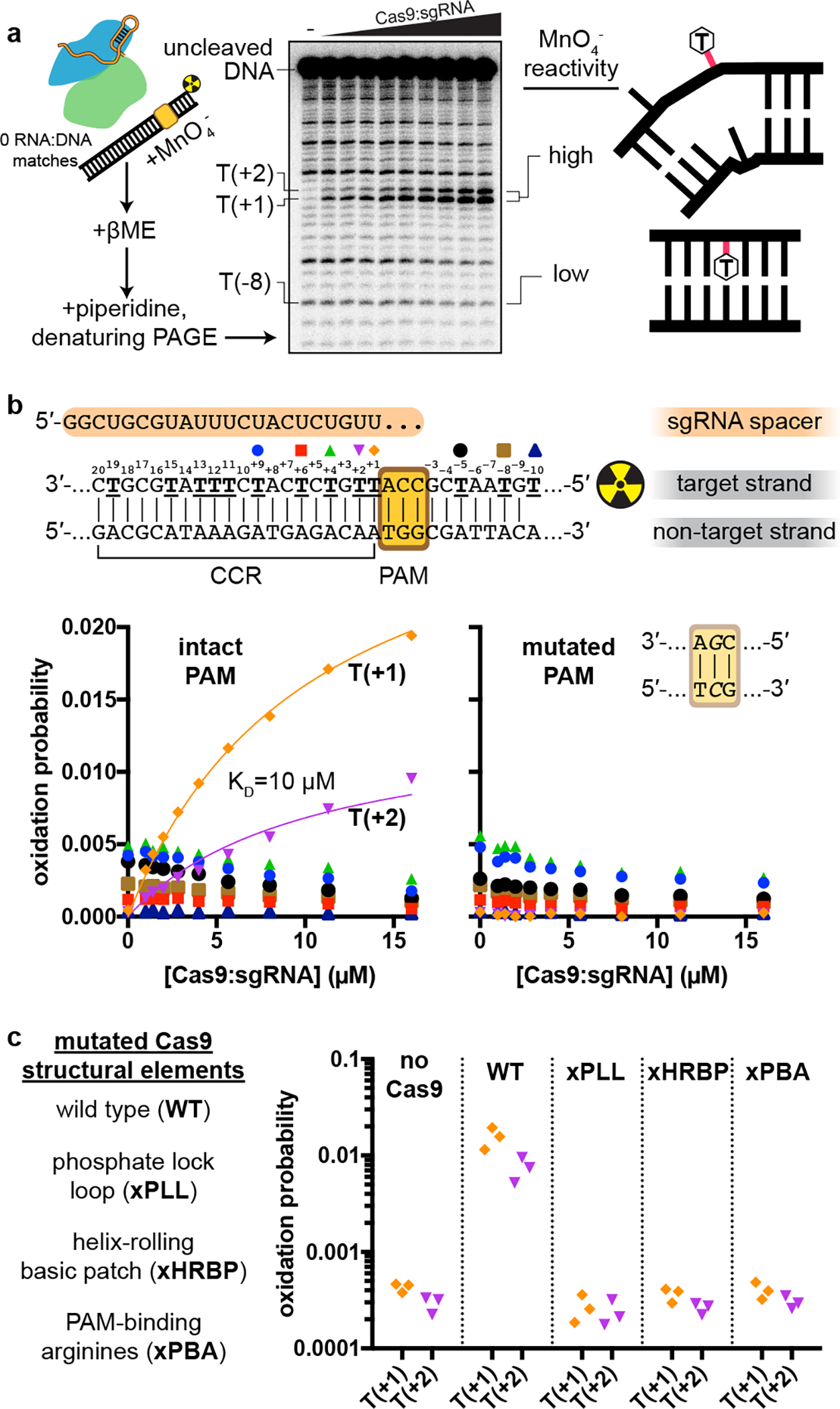
Permanganate reactivity measurements. **a**, Experimental pipeline. The autoradiograph depicts the raw data used to produce the “intact PAM” graph in **b**. **b**, Oxidation probability of select thymines as a function of [Cas9:sgRNA]. Data depict a single replicate. Model information and additional replicates/thymines are presented in Extended Data Fig. 7a. CCR, candidate complementarity region. **c**, Oxidation probability of T(+1) and T(+2) in the presence of the indicated Cas9:sgRNA variant ([Cas9:sgRNA]=16 μM). Three replicates depicted. xPLL, KES(1107–1109)GG; xHRBP, K233A/K234A/K253A/K263A; xPBA, R1333A/R1335A.

Next, we wondered whether local DNA conformations observed in the cross-linked interrogation complex resemble those in the native complex. To characterize DNA distortion with single-nucleotide resolution, we measured the permanganate reactivity of individual thymines in the target DNA strand of a non-cross-linked interrogation complex (Fig. 5a). As anticipated for protein-induced base unstacking^27,28^, we detected a PAM- and Cas9-dependent increase in permanganate reactivity at Thy(+1) and Thy(+2) (Fig. 5a–c, Extended Data Fig. 7a, b). The relationship between permanganate reactivity and Cas9:sgRNA concentration at these thymines suggests that the affinity of Cas9:sgRNA for this sequence is weak (10 μM), as expected for this necessarily transient interaction with off-target DNA (Fig. 5b, Extended Data Fig. 7a). Remarkably, Thy(+1) and Thy(+2) are precisely the nucleotides that appeared to be unpaired in the bent-DNA cryo-EM map of the cross-linked Cas9 interrogation complex (Fig. 3d), which shared the same DNA sequence as the permanganate substrate. These results suggest that Cas9 bends DNA through a backbone distortion that exposes target-strand nucleobases +1 and +2 to solvent and, more generally, that cryo-EM analysis of the cross-linked complex captured meaningful structural features of the native complex.

### A Cas9 conformational rearrangement accompanies DNA bending

The described linear- and bent-DNA conformations present a new model for Cas9 function in which open-protein Cas9:sgRNA first associates with the PAM on linear DNA, then engages a switch to the closed-protein state to bend the DNA and expose its PAM-adjacent nucleobases for interrogation (Supplementary Video 1). Because this transition involves energetically unfavorable base unstacking, we wondered how the unstacked state is stabilized.

**Extended Data Fig. 8:**
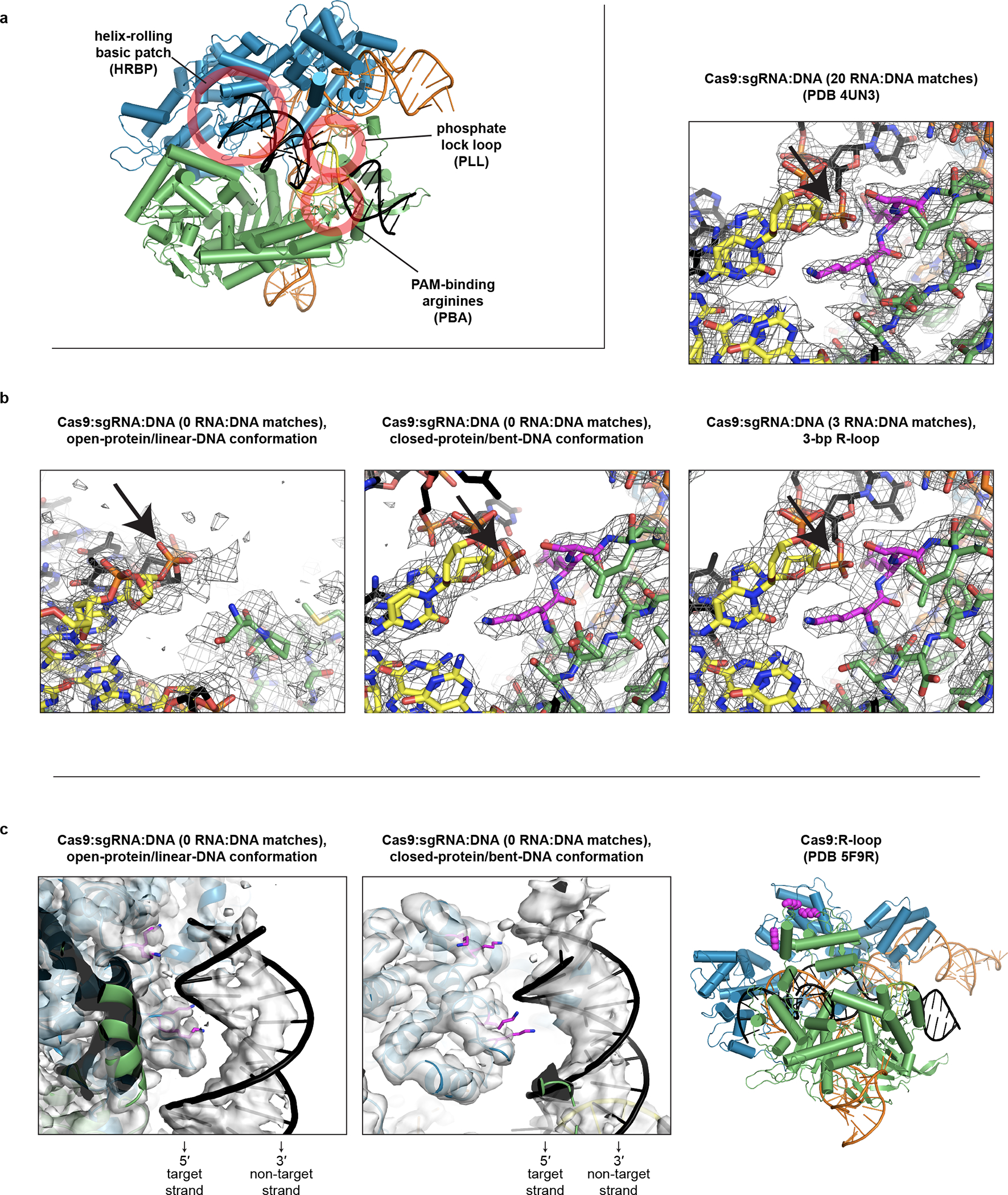
Structural features potentially relevant to Cas9-induced DNA bending. **a**, Location of each feature in the bent-DNA structure. Green, NUC lobe; blue, REC lobe; orange, guide RNA; black, DNA; yellow, PAM. **b**, Comparison of phosphate lock loop (magenta) in various structures, within sharpened cryo-EM maps (row of three, threshold 8σ) or 2Fo-Fc map (upper right, threshold 1.5σ). Black arrow indicates the eponymous phosphate between nucleotides 0 and +1 of the target strand. **c**, Comparison of helix-rolling basic patch (magenta) in various structures, within unsharpened cryo-EM maps (first panel, threshold 5σ; second panel, threshold 6σ).

Remarkably, the “phosphate lock loop” (Lys1107-Ser1109), which was proposed to support R-loop nucleation by tugging on the target-strand phosphate between the PAM and nucleotide +1^13^, is disordered in the linear-DNA structure but stably bound to the target strand in the bent-DNA structure (Extended Data Fig. 8a, b), highlighting this contact as a potential energetic compensator for the base unstacking penalty. In the permanganate assay, mutation of the phosphate lock loop decreased activity to the level observed without Cas9 or with a Cas9 mutant deficient in PAM recognition (which lacks the PAM-binding arginines, “xPBA”) (Fig. 5c, Extended Data Fig. 7b), indicating that the loop may play a role in DNA bending. However, a negative result in this assay could be attributed either to weakened DNA bending activity or to an overall destabilization of the protein:DNA interaction^4^.

Another notable structural element is a group of lysines (Lys233/Lys234/Lys253/Lys263, termed here the “helix-rolling basic patch”) on REC2 (REC lobe domain 2) that contact the DNA phosphate backbone (at bp +8 to +13) in both the linear- and bent-DNA structures, an interaction that has not been observed before (Extended Data Fig. 8a, c). Mutation of these lysines attenuated anisotropy in the cyclization assay (Fig. 4b, Extended Data Fig. 6a) and abolished Cas9’s permanganate sensitization activity (Fig. 5c, Extended Data Fig. 7b). Structural modeling of the linear-to-bent transition (Supplementary Video 2) suggests that the helix-rolling basic patch may couple DNA bending to inter-lobe protein rotations similar to those observed in multi-body refinements^29^ of the cryo-EM images (Supplementary Video 3–4). Consensus EM reconstructions also revealed large segments of the REC lobe and guide RNA that become ordered upon lobe closure (Supplementary Video 1, Supplementary Information), implying that Cas9 can draw upon diverse structural transitions across the complex to regulate DNA bending.

### The bent-DNA state makes R-loop nucleation structurally accessible

**Extended Data Fig. 9:**
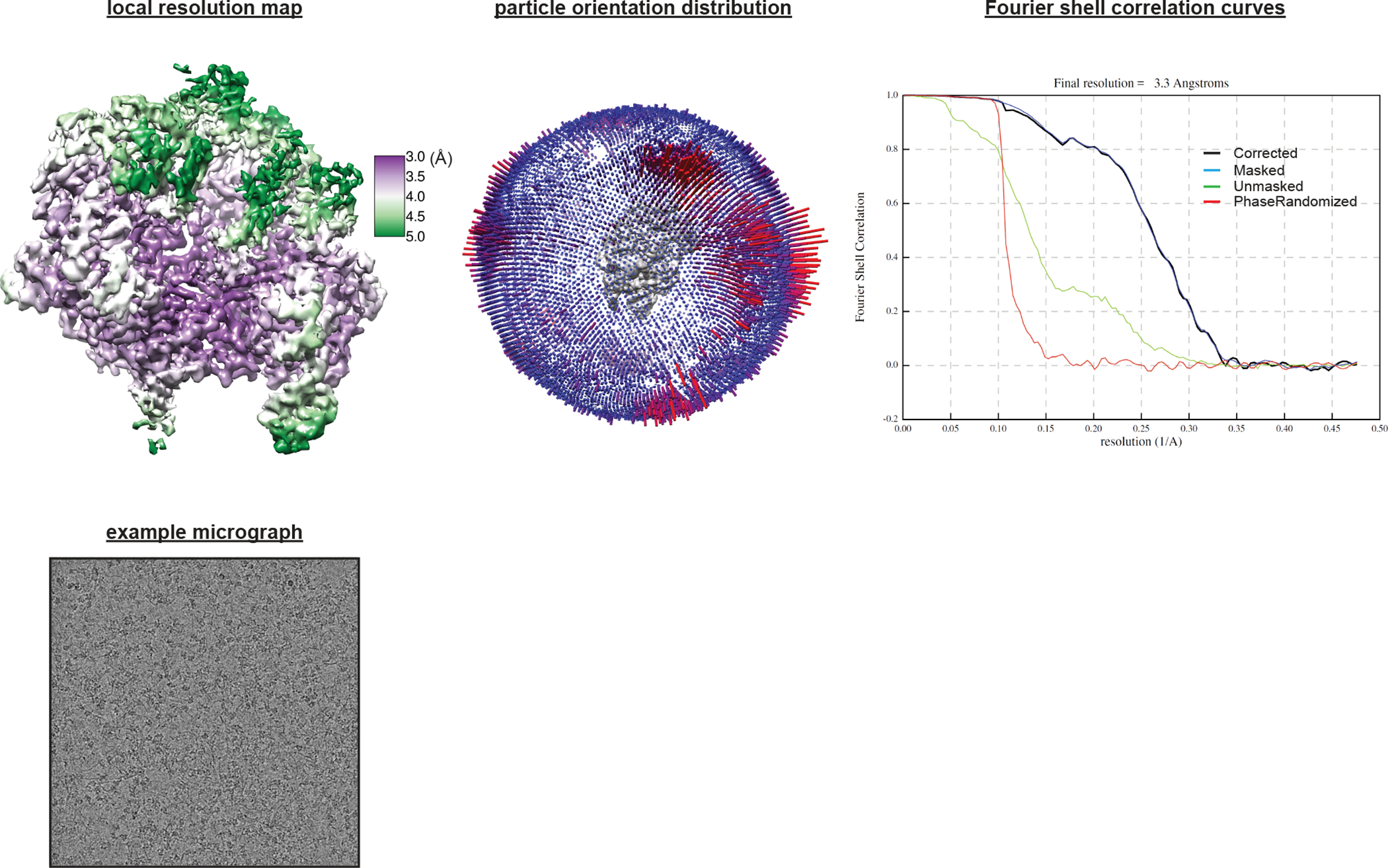
Cryo-EM analysis of Cas9:sgRNA:DNA with 3 RNA:DNA matches. Details of cryo-EM analysis.

**Fig. 6 |:**
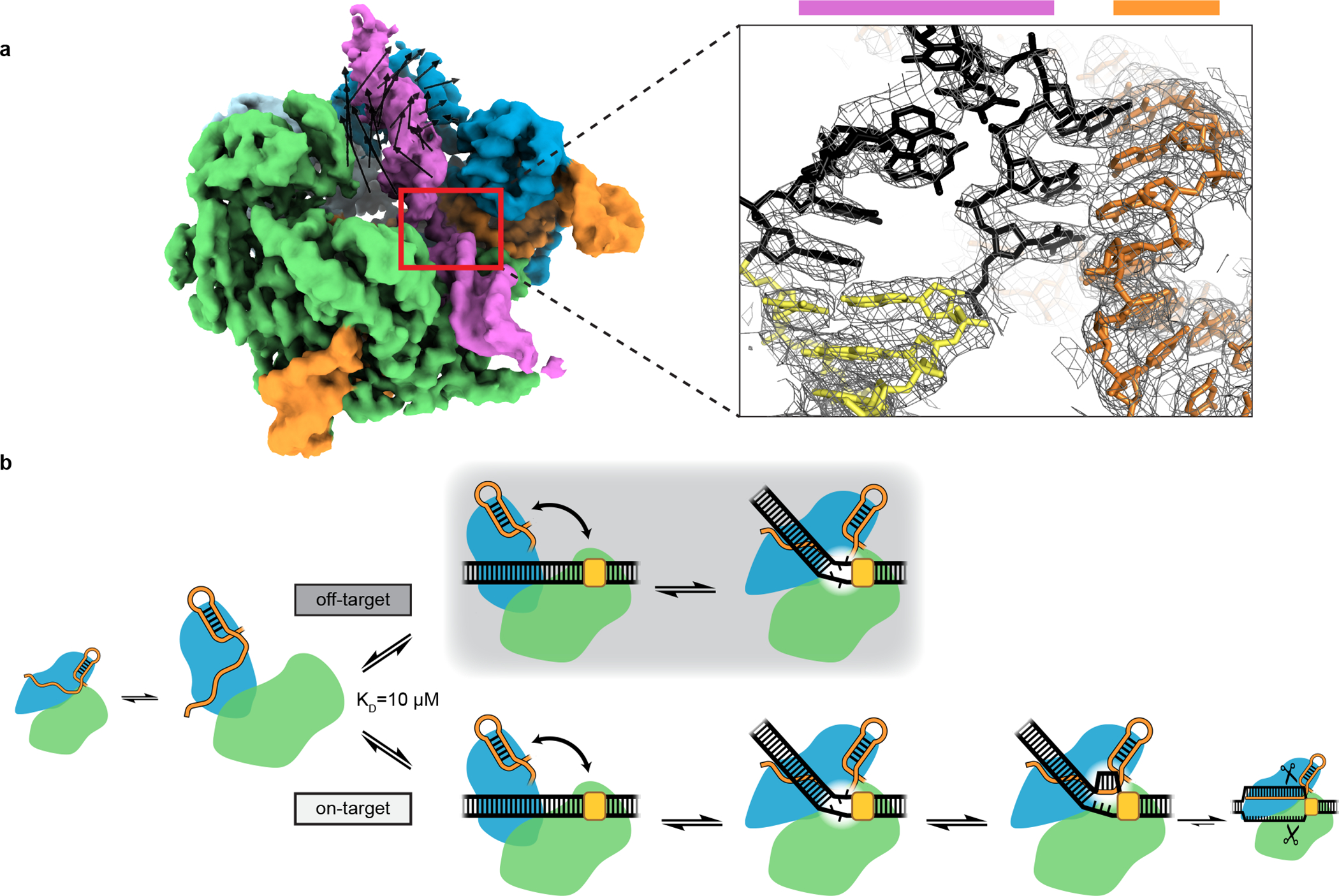
Structure of a nascent R-loop and overview model. **a**, Left, unsharpened cryo-EM map (threshold 5σ) of Cas9:sgRNA:DNA with 3 RNA:DNA bp. Green, NUC lobe; blue, REC lobe; orange, guide RNA; magenta, DNA. Black vectors indicate differences from the bent-DNA structure with 0 RNA:DNA matches. The primary difference is a rigid-body rotation of REC lobe domain 2. Inset, sharpened cryo-EM map (threshold 8σ) and model. For clarity, only DNA and RNA are shown. Black, candidate complementarity region; yellow, PAM. **b**, Model for bend-dependent Cas9 target search and capture. Large diagrams depict states structurally characterized in the present work.

We propose that the function of the bent-DNA conformation is to promote local base flipping that can lead to R-loop nucleation. To probe the structure of a complex that has already proceeded to the R-loop nucleation step, we employed the same cross-linking strategy with adjusted RNA and DNA sequences that allow partial R-loop formation (Fig. 1c). Cryo-EM analysis of this construct revealed nucleotides +1 to +3 of the DNA target strand hybridized to the sgRNA spacer (Fig. 6a, Extended Data Fig. 9). In contrast to the disorder that characterized this region in the bent-DNA map, all three nucleotides are well-resolved, apparently stabilized by their hybridization to the A-form sgRNA spacer. The increase in resolution extends to the non-target strand and to the more PAM-distal regions of the CCR, suggesting that the DNA becomes overall more ordered in response to R-loop nucleation. The ribonucleoprotein architecture resembles that of the bent-DNA structure except for slight tilting of REC2, which accommodates a repositioning of the CCR duplex toward the newly formed RNA:DNA base pairs (Fig. 6a). Therefore, unstacked nucleotides in the bent-DNA state can hybridize to the sgRNA spacer with minimal global structural changes, further supporting the bent-DNA structure as a gateway to R-loop nucleation.

Our structures outline a model for a poorly understood aspect of Cas9 function that is fundamental to CRISPR target search and capture (Fig. 6b). First, open-conformation Cas9:sgRNA associates with the PAM of a linear DNA target. By engaging the open-to-closed protein conformational switch, Cas9 bends and twists the DNA to locally unwind the base pairs next to the PAM. If target-strand nucleotides are unable to hybridize to the sgRNA spacer, the candidate target is released, and Cas9 proceeds to the next candidate. If the target strand is sgRNA-complementary, unwound nucleotides initiate an RNA:DNA hybrid that can expand through strand invasion to a full 20-bp R-loop, activating DNA cleavage.

Due to its energetic linkage to base flipping^30^, DNA bending provides a viable mechanical solution to any biological problem that requires unrestricted access to nucleobases^31,32^, including the sequence interrogation challenge faced by all DNA-targeting CRISPR systems^4,33–36^. In our key structural snapshot of this process, Cas9 specifically employs a bending mode that involves underwinding (Fig. 3), which may be a topological necessity for the downstream propagation of flipping events, and which could underlie some features of Cas9’s mechanical sensitivity^37–39^. In contrast, certain methyltransferases that flip only one nucleotide at a time can afford to do so without gross helical distortion^40,41^.

Interestingly, other proteins define the vertex of an underwound DNA bend using intimate contacts to the distorted nucleotides, either to intact base pairs in the case of transcription factors^18–20,23^ or to flipped bases and their estranged partners in the case of base excision repair enzymes^16,17,21,22,42^. During initial DNA interrogation, Cas9 appears to make no such contacts, instead straddling the bending vertex and relying on mechanical strain to stabilize extrahelical nucleotide conformations without restricting their motion. To catalyze RNA-programmable strand exchange, Cas9 must distort DNA independent of its sequence, likening its functional constraints to those of the filamentous recombinase RecA^43^, which non-specifically destabilizes candidate DNA via longitudinal stretching^44–46^.

The mechanism illustrated here reveals for the first time the individual steps that comprise the slowest phase of Cas9’s genome editing function^11^. The energetic tuning of binding, bending, and RNA:DNA hybrid nucleation dictates the speed of target capture—bent/unstacked states must be stable enough to promote fast transitions to RNA-hybridized states, but not so stable that Cas9 wastes undue time on off-target DNA. Thus, the energetic landscape surrounding the states identified in this work will be a crucial subject of study to understand the success of current state-of-the-art genome editors and to inform the engineering of faster ones. Finally, DNA in eukaryotic chromatin is rife with bends, due either to intrinsic structural features of the DNA sequence^47^ or to interactions with looping proteins^48^. Because the Cas9-induced DNA bend described here has a well-defined direction that may either match or antagonize incumbent bends, it will be important to test how local chromatin geometry affects Cas9’s efficiency in both dissociating from off-target sequences and opening R-loops on real target sequences.

## Methods

### Protein expression and purification

Cas9 was expressed and purified as described previously^28^, with slight modifications. Briefly, protein was expressed from custom pET-based vectors in *E. coli* BL21 Star(DE3) cells. Cells were sonicated in lysis buffer (50 mM HEPES (pH 7.5), 500 mM NaCl, 1 mM TCEP, 0.5 mM PMSF, 10 tablets/L cOmplete EDTA-free protease inhibitor cocktail (Roche), 0.25 mg/mL chicken egg white lysozyme (Sigma-Aldrich)), and clarified lysate was loaded onto Ni-NTA resin, which was then washed (50 mM HEPES (pH 7.5), 500 mM NaCl, 1 mM TCEP, 5% glycerol, 20 mM imidazole) and eluted (50 mM HEPES (pH 7.5), 500 mM NaCl, 1 mM TCEP, 5% glycerol, 300 mM imidazole). Proteins were cleaved overnight with TEV protease at 4°C without dialysis. The digested protein solution was diluted with one volume of low-salt ion exchange buffer (50 mM HEPES (pH 7.5), 250 mM KCl, 1 mM TCEP, 10% glycerol). Digested protein was purified on a HiTrap Heparin HP affinity column (Cytiva), eluting with high-salt ion exchange buffer (50 mM HEPES (pH 7.5), 1 M KCl, 1 mM TCEP, 10% glycerol). Eluted protein was then purified by size exclusion in protein-purification size exclusion buffer (20 mM HEPES (pH 7.5), 150 mM KCl, 1 mM dithiothreitol (DTT), 10% glycerol) on a Superdex 200 Increase 10/300 GL column (Cytiva). Plasmid/protein sequences and Addgene IDs can be found in the Supplementary Information.

### Nucleic acid preparation

All DNA oligonucleotides were synthesized by Integrated DNA Technologies except the cystamine-functionalized target strand, which was synthesized by TriLink Biotechnologies (with HPLC purification). DNA oligonucleotides that were not HPLC-purified by the manufacturer were PAGE-purified in house (unless a downstream preparative step involved another PAGE purification), and all DNA oligonucleotides were stored in water. Duplex DNA substrates were annealed by heating to 95°C and cooling to 25°C over the course of 40 min on a thermocycler. Guide RNAs were transcribed and purified as described previously^28^, except no ribozyme was included in the transcript. Briefly, *in vitro* transcription reactions included PCR-assembled DNA template, 40 mM Tris-Cl (pH 7.9 at 25°C), 25 mM MgCl_2_, 10 mM DTT, 0.01% (v/v) Triton X-100, 2 mM spermidine, 5 mM of each NTP, and 100 μg/mL T7 RNA polymerase. Transcription was allowed to proceed for 2.5 hr at 37°C, after which RNA was purified by urea-PAGE, ethanol-precipitated, and resuspended in RNA storage buffer (0.1 mM EDTA, 2 mM sodium citrate, pH 6.4). All sgRNA molecules were annealed (80°C for 2 min, then moved directly to ice) in RNA storage buffer prior to use. For both DNA and RNA, A_260_ was measured on a NanoDrop (Thermo Scientific), and concentration was estimated according to extinction coefficients reported previously^49^. Oligonucleotide sequences can be found in the Supplementary Information.

### Cryo-EM construct preparation

DNA duplexes were pre-annealed in water at 10X concentration (60 μM target strand, 75 μM non-target strand). Cross-linking reactions were assembled with 300 μL water, 100 μL 5X disulfide reaction buffer (250 mM Tris-Cl, pH 7.4 at 25°C, 750 mM NaCl, 25 mM MgCl_2_, 25% glycerol, 500 μM DTT), 50 μL 10X DNA duplex, 25 μL 100 μM sgRNA, and 25 μL 80 μM Cas9. Cross-linking was allowed to proceed at 25°C for 24 hours (0 RNA:DNA matches) or 8 hours (3 RNA:DNA matches). Sample was then purified by size exclusion (Superdex 200 Increase 10/300 GL, Cytiva) in cryo-EM buffer (20 mM Tris-Cl, pH 7.5 at 25°C, 200 mM KCl, 100 μM DTT, 5 mM MgCl_2_, 0.25% glycerol). Peak fractions were pooled, concentrated to an estimated 6 μM, snap-frozen in 10-μL aliquots in liquid nitrogen, and stored at −80°C until grid preparation. For the Cas9:sgRNA structural construct, which lacked a cross-link, the reaction was assembled with 350 μL water, 100 μL 5X disulfide reaction buffer, 0.45 μL 1 M DTT, 25 μL 100 μM sgRNA, and 25 μL 80 μM Cas9. The complex was allowed to form at 25°C for 30 minutes. The Cas9:sgRNA sample was then size-exclusion-purified and processed as described for the DNA-containing constructs. For Cas9:sgRNA, cryo-EM buffer contained 1 mM DTT instead of 100 μM DTT.

### SDS-PAGE analysis

For non-reducing SDS-PAGE, thiol exchange was first quenched by the addition of 20 mM S-methyl methanethiosulfonate (S-MMTS). Then, 0.25 volumes of 5X non-reducing SDS-PAGE loading solution (0.0625% w/v bromophenol blue, 75 mM EDTA, 30% glycerol, 10% SDS, 250 mM Tris-Cl, pH 6.8) were added, and the sample was heated to 90°C for 5 minutes before loading of 3 pmol onto a 4–15% Mini-PROTEAN TGX Stain-Free Precast Gel (Bio-Rad), alongside PageRuler Prestained Protein Ladder (Thermo Scientific). Gels were imaged using the Stain-Free imaging protocol (5-min activation, 3-s exposure) of Bio-Rad Image Lab 5.2.1 on a Bio-Rad ChemiDoc. For reducing SDS-PAGE, no S-MMTS was added, and 5% β-mercaptoethanol (βME) was added along with the non-reducing SDS-PAGE loading solution. For radioactive SDS-PAGE analysis, a 4–20% Mini-PROTEAN TGX Precast Gel (Bio-Rad) was pre-run for 20 min at 200 V (to allow free ATP to migrate ahead of free DNA), run with radioactive sample for 15 min at 200 V, dried (80°C, 3 hours) on a gel dryer (Bio-Rad), and exposed to a phosphor screen, subsequently imaged on an Amersham Typhoon using the Amersham Typhoon Control Software 2.0.0.6 (Cytiva).

### Nucleic acid radiolabeling

Standard 5′ radiolabeling was performed with T4 polynucleotide kinase (New England Biolabs) at 0.2 U/μL (manufacturer’s units), 1X T4 PNK buffer (New England Biolabs), 400 nM DNA oligonucleotide, and 200 nM [γ−^32^P]-ATP (PerkinElmer) for 30 min at 37°C, followed by a 20-min heat-killing incubation at 65°C. Radiolabeled oligos were then buffer exchanged into water using a Microspin G-25 spin column (GE Healthcare). For 5′ radiolabeling of sgRNAs, the 5′ triphosphate was first removed by treatment with Quick CIP (New England BioLabs, manufacturer’s instructions). The reaction was then supplemented with 5 mM DTT and the same concentrations of T4 polynucleotide kinase (New England BioLabs) and [γ−^32^P]-ATP (PerkinElmer) used for DNA radiolabeling, and the remainder of the protocol was completed as for DNA.

### Radiolabeled target-strand cleavage rate measurements

DNA duplexes at 10X concentration (20 nM radiolabeled target strand, 75 μM unlabeled non-target strand) were annealed in water with 60 μM cystamine dihydrochloride (pH 7). A 75-μL reaction was assembled from 15 μL 5X Mg-free disulfide reaction buffer (250 mM Tris-Cl, pH 7.4 at 25°C, 750 mM NaCl, 5 mM EDTA, 25% glycerol, 500 μM DTT), 7.5 μL 600 μM cystamine dihydrochloride (pH 7), 37.5 μL water, 3.75 μL 80 μM Cas9, 3.75 μL 100 μM sgRNA, 7.5 μL 10X DNA duplex. The reaction was incubated at 25°C for 2 hours, at which point the cross-linked fraction had fully equilibrated. To non-reducing or reducing reactions, 5 μL of 320 mM S-MMTS or 80 mM DTT (respectively) in 1X Mg-free disulfide reaction buffer was added. Samples were incubated at 25°C for an additional 5 min, then cooled to 16°C and allowed to equilibrate for 15 min. One aliquot was quenched into 0.25 volumes 5X non-reducing SDS-PAGE solution and subject to SDS-PAGE analysis to assess the extent of cross-linking (for the reduced sample, no βME was added, as the DTT had already effectively reduced the sample). Another aliquot was quenched for reducing urea-PAGE analysis as timepoint 0. DNA cleavage was initiated by combining the remaining reaction volume with 0.11 volumes 60 mM MgCl_2_. Aliquots were taken at the indicated timepoints for reducing urea-PAGE analysis.

### Urea-PAGE analysis

To each sample was added 1 volume of 2X urea-PAGE loading solution (92% formamide, 30 mM EDTA, 0.025% bromophenol blue, 400 μg/mL heparin). For reducing urea-PAGE analysis, 5% βME was subsequently added. Samples were heated to 90°C for 5 minutes, then resolved on a denaturing polyacrylamide gel (10% or 15% acrylamide:bis-acrylamide 29:1, 7 M urea, 0.5X TBE). For radioactive samples, gels were dried (80°C, 3 hr) on a gel dryer (Bio-Rad), exposed to a phosphor screen, and imaged on an Amersham Typhoon (Cytiva). For samples containing fluorophore-conjugated DNA, gels were directly imaged on the Typhoon without further treatment. For unlabeled samples, gels were stained with 1X SYBR Gold (Invitrogen) in 0.5X TBE prior to Typhoon imaging.

### Fluorescence and autoradiograph data analysis

Band volumes in fluorescence images and autoradiographs were quantified in Image Lab 6.1 (Bio-Rad). For fluorescence images recorded by the ChemiDoc, Image Lab’s native .scn files were used for quantification. For images recorded by the Typhoon, the Typhoon software’s native .gel files (square root encoded) were used for quantification. Data were fit by the least-squares method in Prism 7 (GraphPad Software).

### Cryo-EM grid preparation and data collection

Cryo-EM samples were thawed and diluted to 3 μM (Cas9:sgRNA:DNA) or 1.5 μM (Cas9:sgRNA) in cryo-EM buffer. An UltrAuFoil grid (1.2/1.3-μm, 300 mesh, Electron Microscopy Sciences, catalog no. Q350AR13A) was glow-discharged in a PELCO easiGlow for 15 s at 25 mA, then loaded into an FEI Vitrobot Mark IV equilibrated to 8°C with 100% humidity. From the sample, kept on ice up until use, 3.6 μL was applied to the grid, which was immediately blotted (Cas9:sgRNA:DNA{0 RNA:DNA matches} and Cas9:sgRNA: blot time 4.5 s, blot force 8; Cas9:sgRNA:DNA{3 RNA:DNA matches}: blot time 3 s, blot force 6) and plunged into liquid-nitrogen-cooled ethane. Micrographs for Cas9:sgRNA were collected on a Talos Arctica TEM operated at 200 kV and x36,000 magnification (1.115 Å/pixel), at −0.8 to −2 μm defocus, using the super-resolution camera setting (0.5575 Å/pixel) on a Gatan K3 Direct Electron Detector. Micrographs for Cas9:sgRNA:DNA complexes were collected on a Titan Krios G3i TEM operated at 300 kV with energy filter, x81,000 nominal magnification (1.05 Å/pixel), −0.8 μm to −2 μm defocus, using the super-resolution camera setting (0.525 Å/pixel) in CDS mode on a Gatan K3 Direct Electron Detector. All images were collected using beam shift in SerialEM v.3.8.7 software.

### Cryo-EM data processing and model building

Details of cryo-EM data processing and model building can be found in the Supplementary Information.

### Permanganate reactivity measurements

DNA duplexes were annealed at 50X concentration (100 nM radiolabeled target strand, 200 nM unlabeled non-target strand) in 1X annealing buffer (10 mM Tris-Cl, pH 7.9 at 25°C, 50 mM KCl, 1 mM EDTA), then diluted to 10X concentration in water. A Cas9 titration at 5X was prepared by diluting an 80 μM Cas9 stock solution with protein-purification size exclusion buffer. An sgRNA titration at 5X was prepared by diluting a 100 μM sgRNA stock solution with RNA storage buffer. For all reactions, the sgRNA concentration was 1.25 times the Cas9 concentration, and the reported ribonucleoprotein concentration is that of Cas9. Reactions were assembled with 11 μL 5X permanganate reaction buffer (100 mM Tris-Cl, pH 7.9 at 25°C, 120 mM KCl, 25 mM MgCl_2_, 5 mM TCEP, 500 μg/mL UltraPure BSA, 0.05% Tween-20), 11 μL water, 11 μL 5X Cas9, 11 μL 5X sgRNA, 5.5 μL 10X DNA. A stock solution of KMnO_4_ was prepared fresh in water, and its concentration was corrected to 100 mM (10X reaction concentration) based on 8 averaged NanoDrop readings (ε_526_ = 2.4 × 10^3^ M^−1^ cm^−1^). Reaction tubes and KMnO_4_ (or water, for reactions lacking permanganate) were equilibrated to 30°C for 15 minutes. To initiate the reaction, 22.5 μL of Cas9:sgRNA:DNA was added to 2.5 μL 100 mM KMnO_4_ or water. After 2 min, 25 μL 2X stop solution (2 M βME, 30 mM EDTA) was added to stop the reaction, and 50 μL of water was added to each quenched reaction. Samples were extracted once with 100 μL 25:24:1 phenol:chloroform:isoamyl alcohol (pH 8) in 5PRIME Phase Lock Heavy tubes (Quantabio). The aqueous phase was isolated and combined with 10 μL 3 M sodium acetate (pH 5.2), 1 μL GlycoBlue coprecipitant (Invitrogen), and 300 μL ethanol, and left at −20°C for >2 hr. DNA was precipitated by centrifugation, and supernatant was decanted. A second wash was performed with 500 μL 70% ethanol. Pellets were resuspended in 70 μL 10% piperidine and incubated at 90°C for 30 min. Solvent was evaporated in a SpeedVac (ThermoFisher Scientific). Approximate yield was determined by measuring radioactivity of the pellet-containing tube in a benchtop radiation counter (Bioscan QC-4000), and pellets were resuspended in an appropriate volume of loading solution (50% water, 50% formamide, 0.025% w/v bromophenol blue) to normalize signal across samples prior to resolution by denaturing PAGE and autoradiography.

Because piperidine treatment leads to low levels of cleavage at every nucleotide, the exhaustive single-nucleotide ladder could be used to assign band identities, also confirmed by the dark/light pattern (piperidine-catalyzed cleavage at thymines is less efficient than at other nucleotides in the absence of permanganate modification and more efficient in the presence of permanganate modification). Data analysis was performed as follows: let *v_i_* denote the volume of band *i* in a lane with *n* total bands (band 1 is the shortest cleavage fragment, band *n* is the topmost band corresponding to the starting/uncleaved DNA oligonucleotide). The probability of cleavage at thymine *i* is defined as: $p_{cleave,i}=\frac{v_{i}}{\sum_{j=i}^{n}v_{j}}$. Oxidation probability of thymine *i* is defined as: *p_ox,i_* = *p_cleave,i,+ pm_* − *p_cleave,i,−pm_*, where +*pm* indicates the experiment that contained 10 mM KMnO_4_ and −*pm* indicates the no-permanganate experiment. An extensive description of this type of analysis can be found in ref. ^28^.

### Preparation of DNA cyclization substrates

Each variant DNA cyclization substrate precursor was assembled by PCR from two amplification primers (one of which contained a fluorescein-dT) and two assembly primers. Each reaction was 400 μL total (split into 4 × 100-μL aliquots) and contained 1X Q5 reaction buffer (New England BioLabs), 200 μM dNTPs, 200 nM forward amplification primer, 200 nM reverse amplification primer, 1 nM forward assembly primer, 1 nM reverse assembly primer, 0.02 U/μL Q5 polymerase. Thermocycle parameters were as follows: 98°C, 30 s; {98°C, 10 s; 55°C, 20 s; 72°C, 15 s}; {98°C, 10 s; 62°C, 20 s; 72°C, 15 s}; {98°C, 10 s; 72°C, 35 s}x25; 72°C, 2 min; 10°C, ∞. PCR products were phenol-chloroform-extracted, ethanol-precipitated, and resuspended in 80 μL water. To this was added 15 μL 10X CutSmart buffer, 47.5 μL water, and 7.5 μL ClaI restriction enzyme (10,000 units/mL, New England BioLabs), and digestion was allowed to proceed overnight at 37°C. Samples were then combined with 0.25 volumes 5X native quench solution (25% glycerol, 250 μg/mL heparin, 125 mM EDTA, 1.2 mg/mL proteinase K, 0.0625% w/v bromophenol blue), incubated at 55°C for 15 minutes, and resolved on a preparative native PAGE gel (8% acrylamide:bis-acrylamide 37.5:1, 0.5X TBE) at 4°C. Fluorescent bands, made visible on a blue LED transilluminator, were cut out, and DNA was extracted, ethanol-precipitated, and resuspended in water.

### Cyclization efficiency measurements

Each cyclization reaction contained the following components: 1 μL 10X T4 DNA ligase reaction buffer (New England BioLabs), 2 μL water, 1 μL 10X ligation buffer additives (400 μg/mL UltraPure BSA, 100 mM KCl, 0.1% NP-40), 2 μL 80 μM Cas9 (or protein-purification size exclusion buffer), 2 μL 100 μM sgRNA (or RNA storage buffer), 1 μL 25 nM cyclization substrate, 1 μL T4 DNA ligase (400,000 units/mL, New England BioLabs) (or ligase storage buffer). All reaction components were incubated together at 20°C for 15 minutes prior to reaction initiation except for the ligase, which was incubated separately. Reactions were initiated by combining the ligase with the remainder of the components, allowed to proceed at 20°C for 30 minutes, then quenched with 2.5 μL 5X native quench solution. Samples were then incubated at 55°C for 15 minutes, resolved on an analytical native PAGE gel (8% acrylamide:bis-acrylamide 37.5:1, 0.5X TBE) at 4°C, and imaged for fluorescein on an Amersham Typhoon (Cytiva). Monomolecular cyclization efficiency (MCE) for a given lane is defined as (band volume of circular monomers)/(sum of all band volumes). Bimolecular ligation efficiency (BLE) is defined as (sum of band volumes of all linear/circular n-mers, for n≥2)/(sum of all band volumes). The non-specific degradation products indicated in Extended Data Fig. 6a were not included in the analysis.

## Data availability

All data generated or analyzed during this study are included within this manuscript and its supporting information files except for the cryo-EM data/models, which can be accessed as follows: Cas9:sgRNA:DNA (*S. pyogenes*) with 0 RNA:DNA base pairs, open-protein/linear-DNA conformation (PDB 7S3H, EMD-24823); Cas9:sgRNA:DNA (*S. pyogenes*) with 0 RNA:DNA base pairs, closed-protein/bent-DNA conformation (PDB 7S36, EMD-24817); Cas9:sgRNA (*S. pyogenes*) in the open-protein conformation (PDB 7S37, EMD-24818); Cas9:sgRNA:DNA (*S. pyogenes*) forming a 3-base-pair R-loop (PDB 7S38, EMD-24819). The analyses and figures in this study also draw on previously determined structures with PDB codes 4ZT0, 5FQ5, 5F9R, 4CMP, 1CGP, and 4UN3 as described in the figure legends and the Supplementary Information. For figures containing fluorescence images, autoradiographs, or scatter plots, the original data are available as Source Data files.

## Extended Data

## Supplementary Material

Supplementary Information

Video 1

Video 2

Video 3

Video 4

## Figures and Tables

**Table 1 |:** Cryo-EM data collection, refinement and validation statistics

	Cas9:sgRNA:DNA (S. pyogenes) with 0 RNA:DNA base pairs, open-protein/linear-DNA conformation (EMD-24823) (PDB 7S3H)	Cas9:sgRNA:DNA (S. pyogenes) with 0 RNA:DNA base pairs, closed-protein/bent-DNA conformation (EMD-24817) (PDB 7S36)	Cas9:sgRNA (S. pyogenes) in the open-protein conformation (EMD-24818) (PDB 7S37)	Cas9:sgRNA:DNA (S. pyogenes) forming a 3-base-pair R-loop (EMD-24819) (PDB 7S38)
**Data collection and processing**				
Magnification	x81,000	x81,000	x36,000	x81,000
Voltage (kV)	300	300	200	300
Electron exposure (e–/Å^2^)	50	50	50	50
Defocus range (μm)	−0.8 to −2	−0.8 to −2	−0.8 to −2	−0.8 to −2
Pixel size (Å)	1.05 (nom.) / 0.525 (super-res.)	1.05 (nom.) / 0.525 (super-res.)	1.115 (nom.) / 0.5575 (super-res.)	1.05 (nom.) / 0.525 (super-res.)
Symmetry imposed	C1	C1	C1	C1
Initial particle images (no.)	83,86,564	83,86,564	15,94,370	5,006,233
Final particle images (no.)	5,45,450	18,121	87,130	17,424
Map resolution (Å)	2.5	3.2	3.2	3.3
FSC threshold	0.143	0.143	0.143	0.143
Map resolution range (Å)				
**Refinement**				
Initial model used (PDB code)	4ZT0, 5FQ5, 5F9R, 4CMP	4ZT0, 5FQ5, 5F9R	4ZT0, 5F9R, 4CMP	4ZT0, 5FQ5, 5F9R
Model resolution (Å)	3.0	3.2	5.3	3.3
FSC threshold	0.5	0.5	0.5	0.5
Model resolution range (Å)				
Map sharpening *B* factor (Å^2^)				
Model composition				
Non-hydrogen atoms	9,966	13,821	9,392	13,987
Protein residues	1,030	1,353	1,033	1,353
Nucleotides	76	131	46	139
Ligands	1	1	0	1
*B* factors (Å^2^)				
Protein	56.34	80.16	109.19	85.61
Nucleotide	68.92	104.85	225.8	119.07
Ligand	58.68	89.24	−	70.37
R.m.s. deviations				
Bond lengths (Å)	0.007	0.007	0.006	0.007
Bond angles (°)	1.070	0.980	1.037	0.980
Validation				
MolProbity score	1.42	1.36	1.49	1.33
Clashscore	3.77	2.98	4.66	2.62
Poor rotamers (%)	0.11	0.16	0.11	0.08
Ramachandran plot				
Favored (%)	96.28	96.07	96.29	95.85
Allowed (%)	3.72	3.93	3.71	4.15
Disallowed (%)	0	0	0	0
